# Microbiota in mesenteric adipose tissue from Crohn’s disease promote colitis in mice

**DOI:** 10.1186/s40168-021-01178-8

**Published:** 2021-11-23

**Authors:** Zhen He, Jinjie Wu, Junli Gong, Jia Ke, Tao Ding, Wenjing Zhao, Wai Ming Cheng, Zhanhao Luo, Qilang He, Wanyi Zeng, Jing Yu, Na Jiao, Yanmin Liu, Bin Zheng, Lei Dai, Min Zhi, Xiaojian Wu, Christian Jobin, Ping Lan

**Affiliations:** 1 Department of Colorectal Surgery, The Sixth Affiliated Hospital of Sun Yat-sen University, Guangzhou, 510655 Guangdong China; 2 Guangdong Provincial Key Laboratory of Colorectal and Pelvic Floor Diseases, Guangdong Institute of Gastroenterology, Guangzhou, 510655 Guangdong China; 3 Zhongshan School of Medicine, Sun Yat-sen University, Guangzhou, 510080 Guangdong China; 4 School of Medicine, Sun Yat-sen University, Guangzhou, 510275 Guangdong China; 5 CAS Key Laboratory of Quantitative Engineering Biology, Shenzhen Institute of Synthetic Biology, Shenzhen Institutes of Advanced Technology (SIAT), Chinese Academy of Sciences, Shenzhen, 518055 Guangdong China; 6 Department of Gastroenterology, The Sixth Affiliated Hospital of Sun Yat-sen University, Guangzhou, 510655 Guangdong China; 7 Department of Medicine, Division of Gastroenterology, University of Florida, CGRC, 2033 Mowry Rd, Gainesville, Florida 32610 USA; 8 Department of Infectious Diseases and Pathology, College of Veterinary Medicine, University of Florida, Gainesville, Florida 32610 USA

**Keywords:** Crohn’s disease, Microbiota, Mesenteric adipose tissue, Bacterial translocation

## Abstract

**Background:**

Mesenteric adipose tissue (mAT) hyperplasia, known as creeping fat is a pathologic characteristic of Crohn’s disease (CD). The reserve of creeping fat in surgery is associated with poor prognosis of CD patients, but the mechanism remains unknown.

**Methods:**

Mesenteric microbiome, metabolome, and host transcriptome were characterized using a cohort of 48 patients with CD and 16 non-CD controls. Multidimensional data including 16S ribosomal RNA gene sequencing (16S rRNA), host RNA sequencing, and metabolome were integrated to reveal network interaction. Mesenteric resident bacteria were isolated from mAT and functionally investigated both in the dextran sulfate sodium (DSS) model and in the *Il10* gene-deficient (*Il10*^−/−^) mouse colitis model to validate their pro-inflammatory roles.

**Results:**

Mesenteric microbiota contributed to aberrant metabolites production and transcripts in mATs from patients with CD. The presence of mAT resident microbiota was associated with the development of CD. *Achromobacter pulmonis* (*A. pulmonis*) isolated from CD mAT could translocate to mAT and exacerbate both DSS-induced and *Il10* gene-deficient (*Il10*^−/−^) spontaneous colitis in mice. The levels of *A. pulmonis* in both mAT and mucous layer from CD patients were higher compared to those from the non-CD group.

**Conclusions:**

This study suggests that the mesenteric microbiota from patients with CD sculpt a detrimental microenvironment and promote intestinal inflammation.

**Video abstract**

**Supplementary Information:**

The online version contains supplementary material available at 10.1186/s40168-021-01178-8.

## Background

Crohn’s disease (CD) is characterized by frequent disease recurrence, with proximately 50% of patients requiring repeated surgery in 5 years after surgery [1–3]. Hyperplasia of mesenteric adipose tissue (mAT), known as “creeping fat,” is a pathologic characteristic of CD and is associated with postoperative recurrence [4]. The presence of creeping fat is thought to be predictive of early clinical recurrence after surgery in patients with CD. Importantly, the inclusion of the mesentery in ileocolic resection for CD is associated with reduced surgical recurrence [5], suggesting that the mAT plays an important role in CD pathogenesis.

Although numerous studies have suggested that the gut microbiota dysbiosis was a key feature in CD development [6], the functional role of translocated microbiota to mAT of CD patients is unclear. Previous studies have provided evidences for the presence of bacteria in adipose tissue from chronic inflammation, such as obesity, insulin resistance, and type 2 diabetes in animal models and humans [7–9]. Alteration of intestinal microbiota in high-fat-diet-fed mice associated with transmucosal bacterial translocation to mAT and blood [10, 11]. Human studies have also demonstrated that intestinal permeability is associated with visceral adiposity [12] and metabolic syndrome in obese individuals [13]. A recent study has provided evidence for the presence of bacteria and bacterial DNA in several mAT in metabolic sequelae of obesity [9]. In addition, a study using a small sample size found a microbiotic signature at the phylogenic level within mATs from CD patients [14]. Interestingly, translocation of viable microbiota in human mAT had been demonstrated to polarize macrophages, therefore resulting in the adipogenesis of mAT and driving the formation of creeping fat in CD patients [15]. Nevertheless, it is still a noteworthy question to clarify the role of mAT-associated microbiota in CD development.

Herein, we performed a comprehensive study of mAT integrating multidimensional datasets of microbiome, metabolome, and transcriptome, from a cohort of 48 CD patients and 16 non-CD controls. We isolated mesenteric resident bacteria from CD patients and demonstrated their colitogenic potential using DSS and *Il10*^*−/−*^ mouse models, likely through an impairment of the intestinal barrier.

## Methods

### Study design and sample collection

Forty-eight patients with CD undergoing ileocolectomy surgery were recruited at the Sixth Affiliated Hospital of Sun Yat-Sen University prospectively. Sixteen patients diagnosed with colorectal cancer based on non-specific symptoms, endoscopic and histopathologic findings of CD, were classified as non-CD controls. All patients underwent collection of anthropometric and routine clinical phenotyping. Individuals were excluded if they were unable to or did not consent to provide tissue, had abdominal surgery history, had taken antibiotics medication within 2 weeks, were contraindicated to surgery, had an acute gastrointestinal infection or perforation, were pregnant, had a known bleeding disorder, were diagnosed with end-stage malignancy. After surgery, colonoscopy was performed to assess the endoscopic recurrence according to the Rutgeerts score. Post-operative recurrence was defined by a Rutgeerts score of ≥ i2.

Mesenteric fat samples were collected before opening the intestine to prevent spill-over contamination during surgery. Mesenteric tissue was excised from the hyperplastic mesenterium of the terminal ileum and immediately put in a sterile collection tube and subsequently put on ice. Those mAT from non-CD controls were resected from the terminal ileum, the same location where CD mAT was collected from. Mesenteric tissue from each subject was divided into 4–5 pieces in an anaerobic chamber. Four of them were respectively subjected for 16S rRNA gene sequencing, metabolic analysis, RNA sequencing, and microbial isolation.

### Isolation and identification of mesenteric bacteria

For the isolation of mesenteric bacteria, fresh resected tissues were chopped and homogenized as previously described [16]. After serial dilutions, the supernatant was spread onto brain heart infusion (BHI) agar plate, BHI-supplemented agar plate, tryptose soya agar (TSA) plate or MacConkey agar plate, and incubated overnight under aerobic or anaerobic conditions for 72 hours (80% N_2_, 10%H_2_, 10%CO_2_), respectively. Bacterial DNA was extracted from each single colony and the identity of individual isolates was verified by Sanger sequencing of the V1-V9 regions of 16S rRNA gene. The following primer set was used for amplifications: 27F 5′-AGAGTTTGATCCTGGCTCAG-3′ and 1492R 5′-GGTTACCTTGTTACGACTT-3′. 16S rRNA sequencing was performed using the Illumina MiSeq platform.

### Mouse experiment

All specific pathogen-free (SPF) mice (male, 6~8 weeks old) were treated with a broad-spectrum antibiotic cocktail (ampicillin 0.2 g/L, metronidazole 0.2 g/L, Neomycin 0.2 g/L, vancomycin 0.1 g/L) in the drinking water for 4 days. For mesenteric pathobiont colonization experiments, mice were orally gavaged with pathobionts (1×10^9^ CFU/dose) every day. All mice were challenged with 3% DSS for 7 days followed by four days of regular drinking. The animals were monitored for weight loss, stool consistency, and hemoccult during the course of experiments, and these parameters were used to compute the disease activity index (DAI). Mice were sacrificed on day 16 and their colon tissues were fixed in 4% paraformaldehyde for HE staining or modified Carnoy’s fixative for Alcian staining. For the experiment using *Il10*^*−/−*^ mice model, mice were treated with the same antibiotics cocktail and subsequently gavaged with pathobionts (1 × 10^9^ CFU/dose) for 3 weeks. DAI and histological analysis were performed as in previous studies [17, 18].

### Cell culture

The murine cell lines RAW264.7, 3T3-L1, and rat cell line IEC6 cells were purchased from American Type Culture Collection (ATCC). All of the cells were cultured at 37 °C in Dulbecco’s modified Eagle’s medium (DMEM; Gibco, Thermo Fisher Scientific, St Peters, MO, USA) supplemented with 10% fetal bovine serum (FBS; Gibco, Thermo Fisher Scientific, St Peters, MO, USA) in a 5% CO_2_ atmosphere.

### In vitro stimulation by bacteria

Murine macrophage cell lines RAW264.7 and isolated mesenteric bacteria were co-cultured with a multiplicity of infection (MOI) of 10 at 37 °C, 5% CO_2_ for 1 h. Gentamycin (100 μL/ml) and kanamycin (100 μL/ml) were then added and the cells were further cultured for 1 h. For bacteria-cell co-culture experiments, pre-adipocytes 3T3L-1 or epithelial cells IEC6 were co-cultured with *Achromobacter pulmonis* (MOI = 20) at 37 °C, 5% CO_2_ for 4 h. Kanamycin (100 μL/ml) was then added and cells were further cultured for 1 h. RNA were extracted from the infected cells and cytokines were measured by qPCR. For cytotoxicity analysis, cells were infected with bacteria with a MOI of 20 at 37 °C, 5% CO_2_ for 24 h. The release of lactate dehydrogenase (LDH) was evaluated by an LDH Cytotoxicity Assay kit (Beyotime, China).

### Quantification and statistical analysis

Unless otherwise stated in individual method sections above, all statistical analyses were performed using Prism 8 (GraphPad Software, San Diego, CA). Two-tailed Student’s *t*-test (parametric) or Mann–Whitney *U* test (non-parametric). For a comparison of more than 3 groups, statistical analysis was performed using one-way ANOVA (parametric) or Kruskal–Wallis test (non-parametric). All *p* values were two-sided and an adjusted *p* value < 0.05 was considered statistically significant. Details of the statistical tests used and pooled values for several biological replicates are indicated in the respective figure legends. The detailed methods of the procedures are provided in Supplementary material.

## Results

### Flow chart of sample collection and analysis

In order to elucidate a potential interaction between mesenteric adipose tissue (mAT) microbiome and host responses in CD patients, a prospective cohort of 48 patients was recruited consisting of individuals with diagnosed CD. For non-CD controls, we recruited 16 patients diagnosed with colorectal cancer but CD free based on non-specific symptoms, endoscopic and histopathologic findings. All mAT from the terminal ileum were resected under sterile conditions and further assessed using different omics datasets: 16S rRNA gene sequencing, human RNA sequencing (RNA-seq), metabolomes, isolation, and culture (Fig. S1). After surgery, colonoscopy was performed to assess the endoscopic recurrence according to the Rutgeerts score.

### Differential mAT microbiome associates with changes in host transcriptome and metabolome in CD patients

**Fig. 1:**
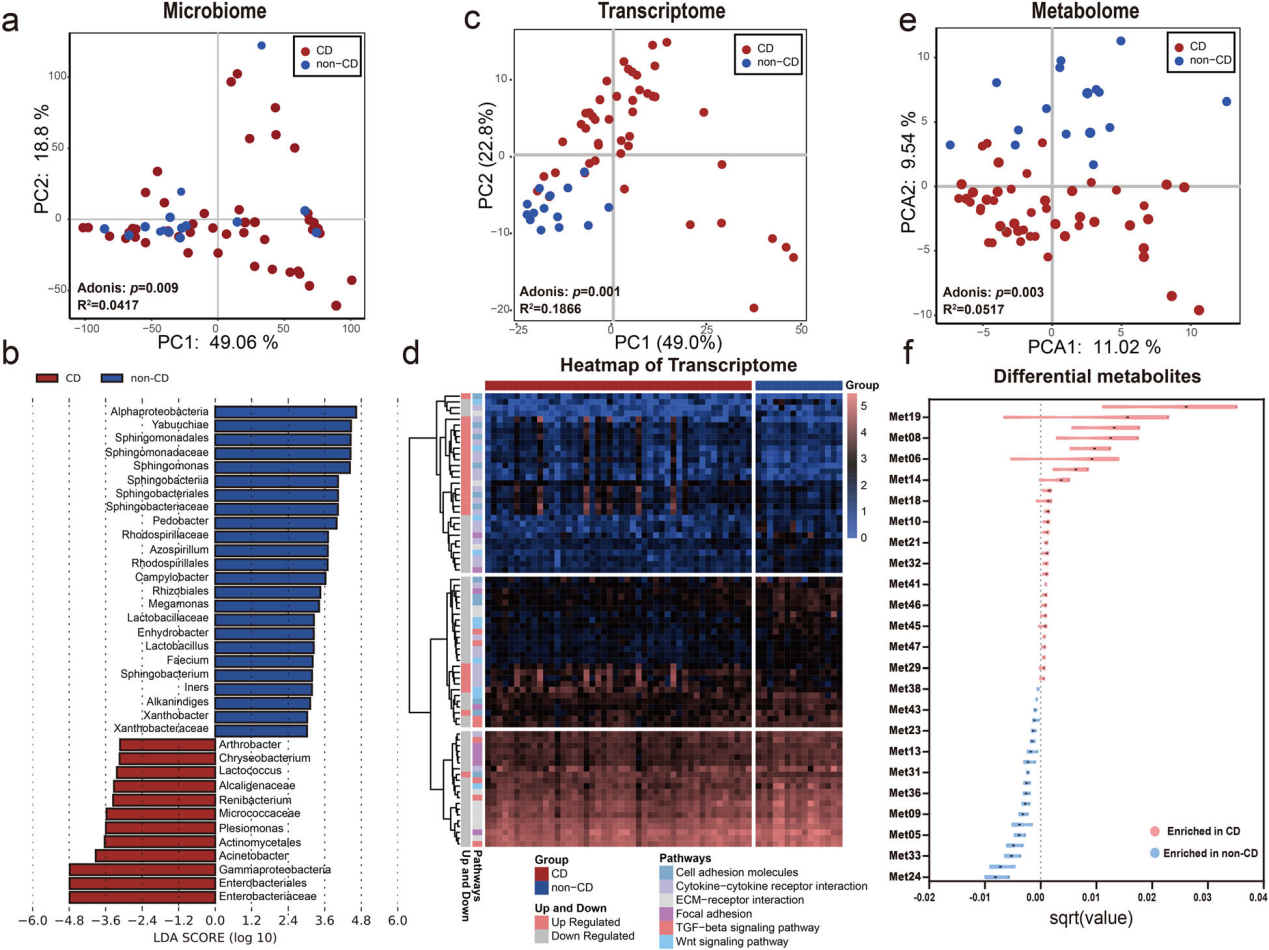
Multi-omics signatures of mesenteric adipose tissue in CD versus non-CD. **a** Separation of mesenteric microbiome between patients with 48 CD and 16 non-CD controls, revealed by principal component analysis (PCA) (Adonis *p* = 0.009). **b** LDA score computed from features differentially abundant between CD patients and non-CD controls. The criteria for feature selection is log LDA score > 3. **c** Separation of the mesenteric transcriptome between 46 CD patients and 15 non-CD controls, revealed by PCA (Adonis *p* = 0.001). **d** Heatmap of the immune-associated DEGs (FDR *p* < 0.05) from 46 patients with CD and 15 non-CD controls. Ordering by diagnosis, clustering within diagnosis. **e** Separation of mesenteric metabolome between 48 CD patients and 16 non-CD controls, revealed by PCA (Adonis *p* = 0.003). **f** Twenty-eight metabolites were significantly (Studentʼs *t*-test, *p* < 0.05) enriched in mAT from 48 CD, while 19 metabolites were significantly enriched in 16 non-CD controls. The violin plot indicated the difference of mean and 95% confidence interval for an individual metabolite. The statistical significance values are denoted as *p < 0.05, **p < 0.01, ***p < 0.001, **** *p* < 0.0001

To exclude the reagent and laboratory contamination, 4 negative sequencing controls (tissue-free blanks processed with the same DNA extraction and PCR amplification and sequenced on the same run) were introduced as quality controls. As expected, no bacterial DNA was detected in quality controls, which was significantly separated from the mAT from patients (Fig. S2a). Taxonomic analysis of mAT microbiome using principal component analysis (PCA) and principal coordinates analysis (PCoA) of 16S rRNA gene sequencing data showed that the mesenteric microbiome from CD and non-CD controls exhibited slight but significant separation (Fig. 1a and Fig. S2b). The α-diversity of microbiome was similar between mAT from CD and the non-CD controls (Fig. S2c and d). Then, we applied linear discriminant analysis of effect size (LEfSe) to detect marked difference in the predominance of bacterial communities between CD and non-CD control. Multiple families such as Enterobacteriaceae, Micrococcaceae, and Alcaligenaceae were enriched in mAT from CD patients, while the families such as Sphingomonadales, Sphingobacteriaceae, and Rhodospirillales were enriched in those from non-CD controls (Fig. 1b). To further clarify the microbial differences, we compared the bacteria at the genus level. As shown in Fig. S2e and f, twenty genera were significantly upregulated in mAT from CD, while five genera were significantly upregulated in non-CD controls. These results revealed the presence of a unique microbial signature in mAT from CD patients.

To further explore the role of the unique mAT microbiota in host alteration, mesenteric tissue (CD: *n* = 46, non-CD: *n* = 15; see “Methods”) were incorporated into our analysis of the host transcriptome. The pattern of transcriptomic features significantly separated CD subjects from non-CD controls (Fig. 1c). We subsequently identified the significantly differentially expressed genes (DEGs) (FDR < 0.05), which were comprised of 512 upregulated and 229 downregulated DEGs. Several DEGs clustered in pathways of extracellular matrix (ECM)-receptor interaction and focal adhesion were significantly downregulated in mAT from CD patients (Fig. 1d), which was consistent with the previous study [15]. Strikingly, the variance of transcriptome between CD and non-CD controls was tightly coupled with the changes of microbiome (Fig. S3a and b). Differential mesenteric microbiota between CD and non-CD controls also exerted significant effects on the variance of the mesenteric transcriptome (Fig. S3c). These findings illustrated that variance of mesenteric microbiota in mAT of CD patients was associated with the alteration of host transcriptome.

The difference in mAT between patients with CD and those non-CD was also apparent at the metabolome level (CD: *n* = 48, non-CD: *n* = 16) (Fig. 1e). Out of all annotated mesenteric metabolites, 28 mesenteric metabolites were significantly elevated in CD relative to non-CD controls, while 19 were significantly depleted in CD patients (Fig. 1f and Fig. S4a). Detailed information about these differential metabolites were shown in Table S1. These differential metabolites were significantly correlated with the predominant bacterial communities in mAT (Fig. S4b). Similarly, the microbes with a significant difference were also associated with the variance of metabolome in mAT (Fig. S4c). These results indicated that a specific pattern of metabolites in mAT from CD patients might be caused by the changes in microbiota. To further dissect interactions between host and microbiota that might underlie mesenteric features in CD, we constructed a large-scale network that incorporated microbiome, differential metabolites, and immune-associated DEGs. Although strong connections were identified in both CD and non-CD groups, the correlation network in mAT from CD was clearly different from that of non-CD controls (Fig. S5a and Table S2).

To better assess the interplay between microbiome, host transcriptome, and metabolome, we incorporated clinical variables prospectively assembled from our cohort. In addition to gender, age, and diagnosis, contemporaneously collected information for each patient included disease type, disease activity, Limberg score, medication history (steroid, mesalamine, and immunotherapy), and trajectories of disease progression with blood parameters (Table S3). Although patients with CD were significantly younger than controls, the age of patients did not significantly affect any omics datasets. Strikingly, the clinical variables associated with CD disease, such as group (CD or non-CD), disease type (locations of inflammation in CD group), and Limberg score, as well as disease activity explained significant effects of the variance for those 3 omics datasets (Fig. S5b). Therefore, our data suggested that alteration of host transcriptome and host-derived metabolites were associated with the changes of microbiota in mAT, which was correlated with CD pathogenesis.

### Presence of Proteobacteria in mAT is associated with the development of CD

**Fig. 2:**
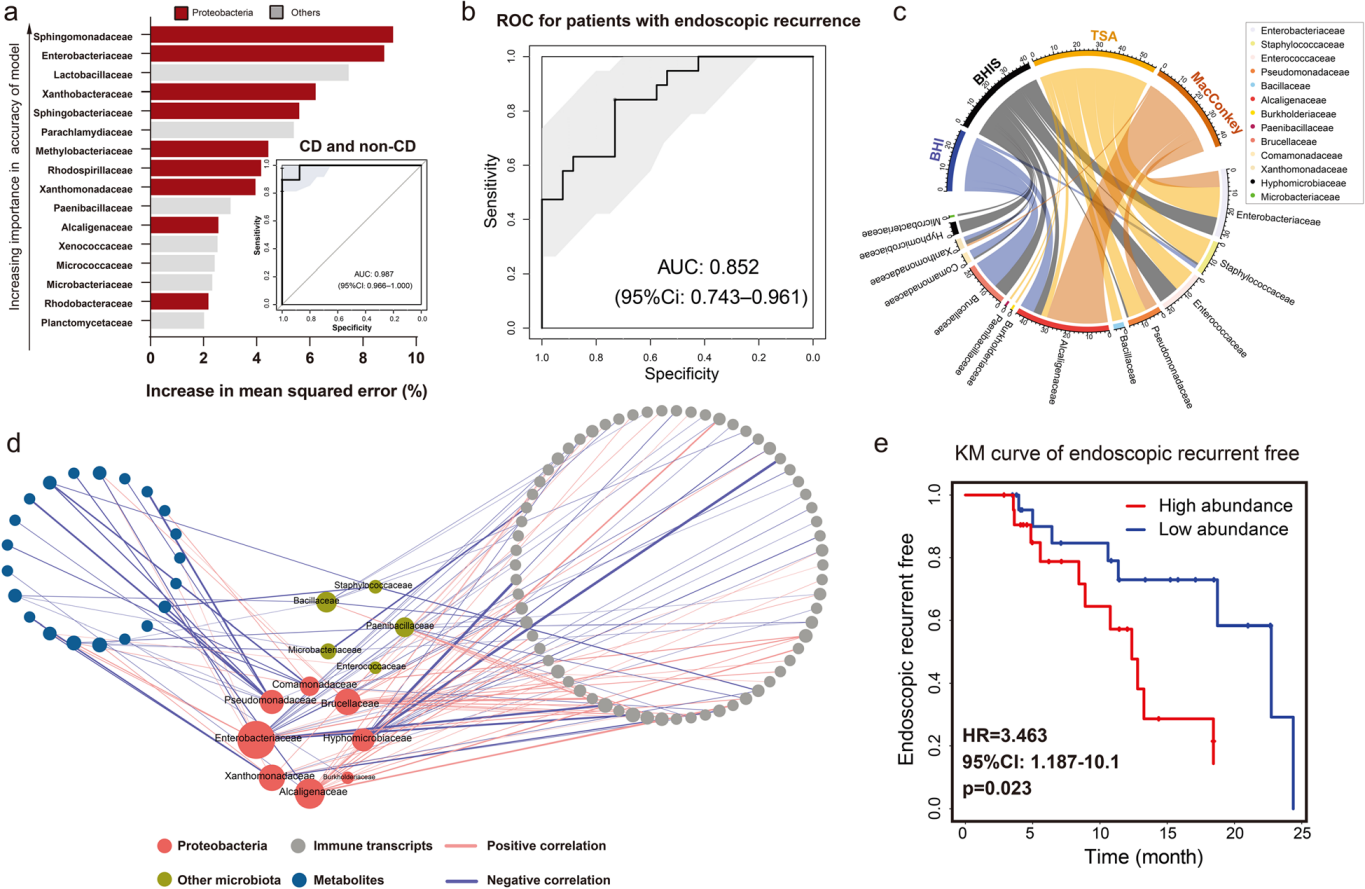
Critical role of isolated bacterial biomarkers in CD inflammation. **a** The top 16 mAT bacterial biomarkers were identified by applying Random Forests regression of their relative abundance. Biomarker taxa are ranked in decreasing order of importance to the accuracy of the model. The model based on these biomarkers achieved an AUC of 0.987. **b** ROC analysis of these mAT bacterial biomarkers in the RF model achieved an AUC of 0.852 to classify patients with endoscopic recurrence. **c** Circularized plot showing the abundance of mesenteric resident bacteria isolated from different culture mediums. **d** Significant association of host-microbiome interactions: isolated species, immune transcripts, and differential metabolites. Network shows the significant correlations (Spearman’s correlation, *p* < 0.05) between two omics variables. Nodes are colored by different omics variables and sized by the number of connections. **e** Kaplan–Meier (KM) survival curves classify patients into high- and low-risk groups based on the major pathogens in the CD cohort

To evaluate the clinical significance of the mAT-associated microbiome in CD, we next applied random forest (RF) and LASSO logistic regression models to determine the role of microbiota in CD. The RF model that maximized the strength of the CD prediction identified 16 important families in mAT (Fig. 2a and Fig. S6a). The model based on these important families exhibited an accuracy of 0.91 for CD/non-CD stratification (Fig. S6b), which achieved an area under the receiver-operating characteristic (ROC) curve (AUC) of 0.986 to distinguish a patient with CD or non-CD (Fig. 2a). Interestingly, half of these important units belonged to the Proteobacteria phylum (Fig. S6c). Importantly, the microbiome selected from the RF model also exhibited an outstanding performance to predict post-operative endoscopic recurrence in CD patients (AUC = 0.852) (Fig. 2b). Similar results were obtained using the LASSO logistic regression classifier (shown in Fig. S6d-g), with an excellent ROC of 0.99 to detect CD patients and an ROC of 0.835 to predict those with endoscopic recurrence (Fig. S6h). These results implicated the important role of mAT-associated microbiota in CD development.

To isolate mAT resident bacteria, we anaerobically and aerobically cultured the mesenteric tissue using different culture mediums (Fig. 2c). Specifically, 174 colonies were picked from the various culture mediums and identified by 16S rRNA gene sequencing, leading to the identification of 32 species belonging to 13 different families presented in mAT from CD (Fig. 2c and Table S5). Consistent with our results described above, eight of these 13 families belonged to the Proteobacteria phylum. We next carried out correlation analysis of these candidate bacteria with metabolites and immune-associated DEGs. Four families exclusive to CD patients and coexisting in the same mAT, including Alcaligenaceae, Brucellaceae, Pseudomonadaceae, and Hyphomicrobiaceae, exhibited a significantly co-varying relationship with a variety of host transcripts and metabolites (Fig. 2d and Fig. S7). Thus we identified 5 candidate strains (5-mix), including *Achromobacter pulmonis (A. pulmonis* from Alcaligenaceae*)*, *Ochrobactrum anthropi (O.anthropi* from Brucellaceae*)*, *Pseudomonas alcaliphila (P.alcaliphila* from Pseudomonadaceae*)*, *Achromobacter deleyi (A.deleyi* from Alcaligenaceae*)*, and *Devosia riboflavina (D.riboflavina* from Hyphomicrobiaceae*)* (Table S5) to represent the major pathogenic members of mesenteric microbiota. In addition, a higher abundance of these 4 candidate families of bacteria in mAT was significantly associated with a higher risk of CD patients with endoscopic recurrence (Fig. 2e), indicating the clinical significance of these bacteria in colitis development.

### Colonization of a defined mesenteric resident bacteria consortium exacerbated colitis in mice

**Fig. 3:**
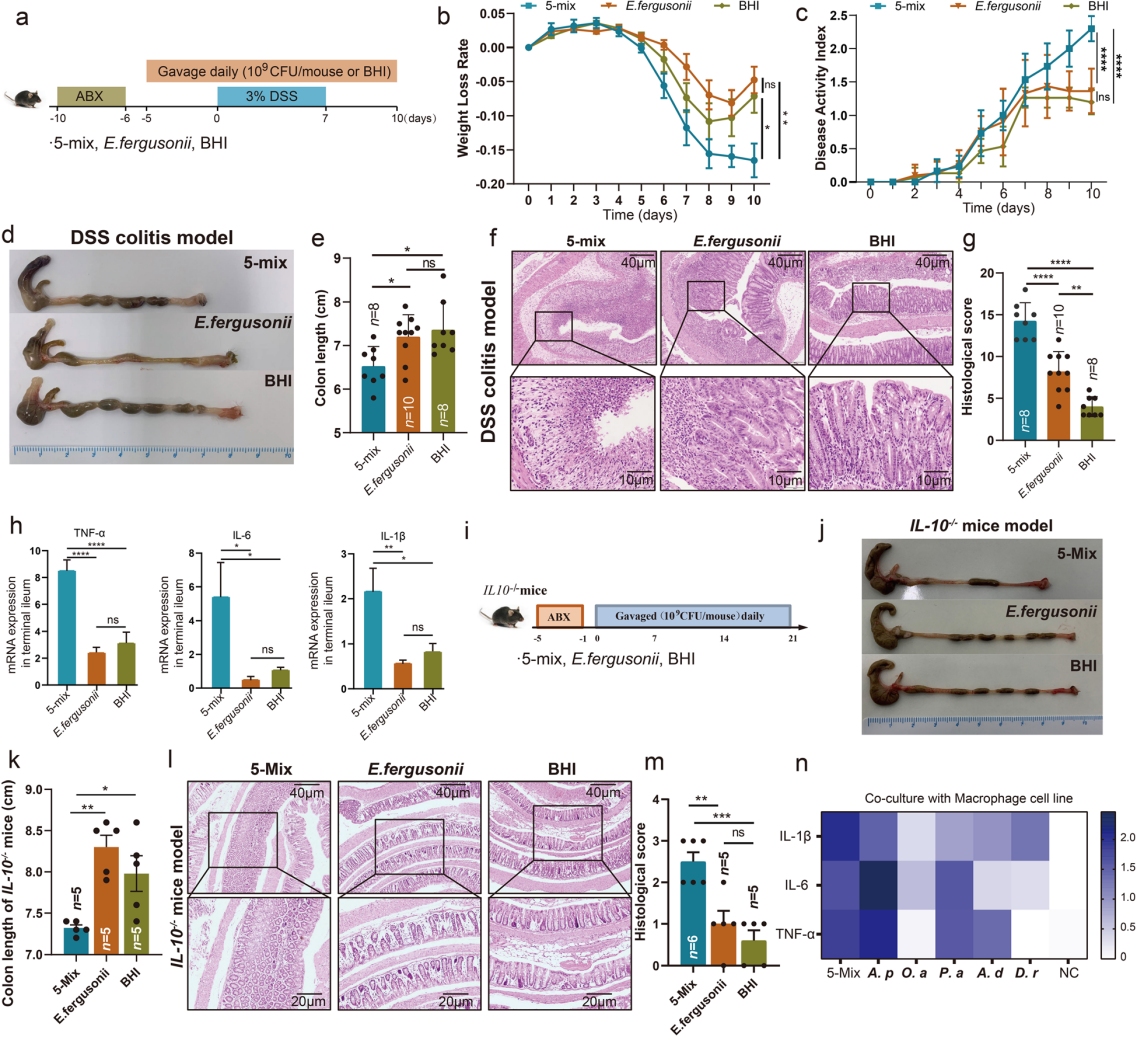
Mesenteric resident bacteria exacerbate colitis in mice. **a** SPF C57BL/6 mice were treated by an antibiotic cocktail for 4 days. One day post antibiotics, the mice were daily orally colonized with bacterial cocktail (5-Mix or *E. fergusonii*, 10^9^ CFU/mouse/dose) or BHI until being euthanized. After 5 days bacterial colonization, the mice were exposed to 3% DSS for 7 days, followed by regular water for 3 days. **b and c** Changes of body weight (**b**) and disease activity index (DAI) after administration of 3% DSS (**c**). **d–g** Representative colons (**d**), colon length (**e**), representative colonic histological images (Scale bar = 40 μm and 10 μm) (**f**), colonic histological score (**g**) in mice treated with 5-mix bacterial cocktail, *E. fergusonii* and BHI. **h** Messenger RNA levels of key cytokines (TNF-α, IL-6, and IL-1β) in the terminal ileum (n=5 per group). **i** SPF *Il10*^*−/−*^ mice were treated with antibiotics cocktail for 4 days. One day post antibiotics, the mice were daily orally colonized with bacterial cocktail (5-Mix or *E. fergusonii*, 10^9^ CFU/mouse/dose) or BHI for 3 weeks. **j–m** Representative colons (**j**), colon length (**k**), representative colonic histological images (Scale bar= 40 μm and 20 μm) (**l**), colonic histological score from *Il10*^*−/−*^ mice treated with 5-Mix, *E. fergusonii* and BHI (**m**). **n** Inflammatory cytokines from macrophage cell line RAW264.7 after co-culture with 5 different bacteria or 5-Mix. Results are shown as the mean ± SEM. Each dot indicates an individual mouse. The statistical significance values are denoted as: *p < 0.05, **p < 0.01, ***p < 0.001, ****p < 0.0001. One-way ANOVA following Tukey’s multiple comparison test (**e**, **g**, **h, k, m,** and **n**); two-way ANOVA following Tukey’s multiple comparison test (**b** and **c**). *A.p*, *A. pulmonis*; *O.a*, *O.anthropi*; *P.a*, *P.alcaliphila*; *A.d*, *A.deleyi*; *D.r*, *D.riboflavina*

To functionally link these mesenteric resident bacteria to intestinal inflammation, we colonized antibiotic-treated specific pathogen-free (SPF) mice with our 5 candidate strains (5-mix) and examined effects on DSS-induced colitis (Fig. 3a). Mice colonized with the commensal bacteria, *Escherichia fergusonii*, isolated from SPF wide-type C57BL/6J mice, were used as controls, which has been demonstrated to be not virulent in a mouse model [19, 20]. These 5 bacteria were capable of colonizing the intestinal lumen as measured by 16S rRNA PCR amplification (Fig. S8a-f). The body weight of mice in those 3 groups was comparable before DSS administration (Fig. S8a). DSS-mediated body weight loss was significantly exacerbated in mice colonized with 5-mix bacteria compared to mice colonized with *E. fergusonii* or culture media alone (BHI) (Fig. 3b). Additionally, DSS-induced colitis as measured by disease activity index (DAI) and colon length was significantly augmented in mice colonized with 5-mix bacteria compared to mice colonized with *E. fergusonii* or BHI (Fig. 3c–e). These clinical parameters correlated with exacerbated histological assessment of colonic inflammation characterized by increased mucosal erosion, crypt destruction, and inflammatory cell infiltration in the mice colonized with 5-Mix bacteria compared with mice colonized with *E. fergusonii* or BHI group (Fig. 3f and g). Consistent with the histological alteration, colonic mRNA expression of TNF-α, IL-6 and IL-1β were significantly elevated in mice colonized with 5-mix bacteria (Fig. 3h). Results were validated in a spontaneous colitis *Il10*^*−/−*^ mouse model (Fig. 3i). Mesenterium-resident bacteria significantly induced shortening (Fig. 3j and k) and destruction of colon tissue (Fig. 3l and m). Collectively, our results demonstrated that the consortium of 5 mesenteric resident bacteria exacerbated DSS-induced colitis.

As our previous transcriptomic analysis indicated the downregulation of ECM-receptor interaction and focal adhesion in mAT, we subsequently evaluated these transcripts in mice treated with 5-mix bacteria. Several transcripts associated with focal adhesion, such as *Egfr*, *Itga9*, *Lama2*, *Pdgfra*, and *Thbs2*, were significantly downregulated in mAT from mice treated with 5-mix bacteria (Fig. S8g). In addition, mRNA expression of genes associated with ECM-receptor interaction including *Col1a1*, *Col 6a1*, *Col 6a2*, *Col 6a3*, *FN1*, and *Tnxb* were decreased in mAT from mice treated with 5-mix bacteria (Fig. S8h). Interestingly, these transcriptional changes were similar to those observed in CD patients. These data implicated the impact of these 5 bacteria on the remodeling of mesenteric structure.

To examine the in vitro inflammatory potential of the 5-mix as well as contribution of individual bacteria, we co-cultured these bacteria with murine RAW264.7 macrophages. Consistent with the results observed in mice, the 5-mix bacteria induced significant secretion of inflammatory cytokines (IL-1β, IL-6, and TNF-α) from macrophages. Although the inflammatory capacity varied among the individual bacterium, we observed that the *Achromobater pulmonis* (*A. pulmonis*) exhibited a significant effect in pro-inflammatory response (Fig. 3n). The secretory pattern of inflammatory cytokines from *A. pulmonis*- treated macrophages was even similar to those treated with 5-mix (Fig. 3n). In addition, *A. pulmonis* also demonstrated strong inducibility of cytotoxicity activity in pre-adipocytes 3T3-L1 or epithelial cell IEC6 by detection of lactate dehydrogenase (LDH) (Fig. S9a and b). We therefore hypothesized that the *A. pulmonis* strain isolated from mAT of CD patients might be the major contributor of exacerbated colitis in vivo.

### Mesenteric resident *Achromobacter pulmonis* exacerbates colitis

**Fig. 4:**
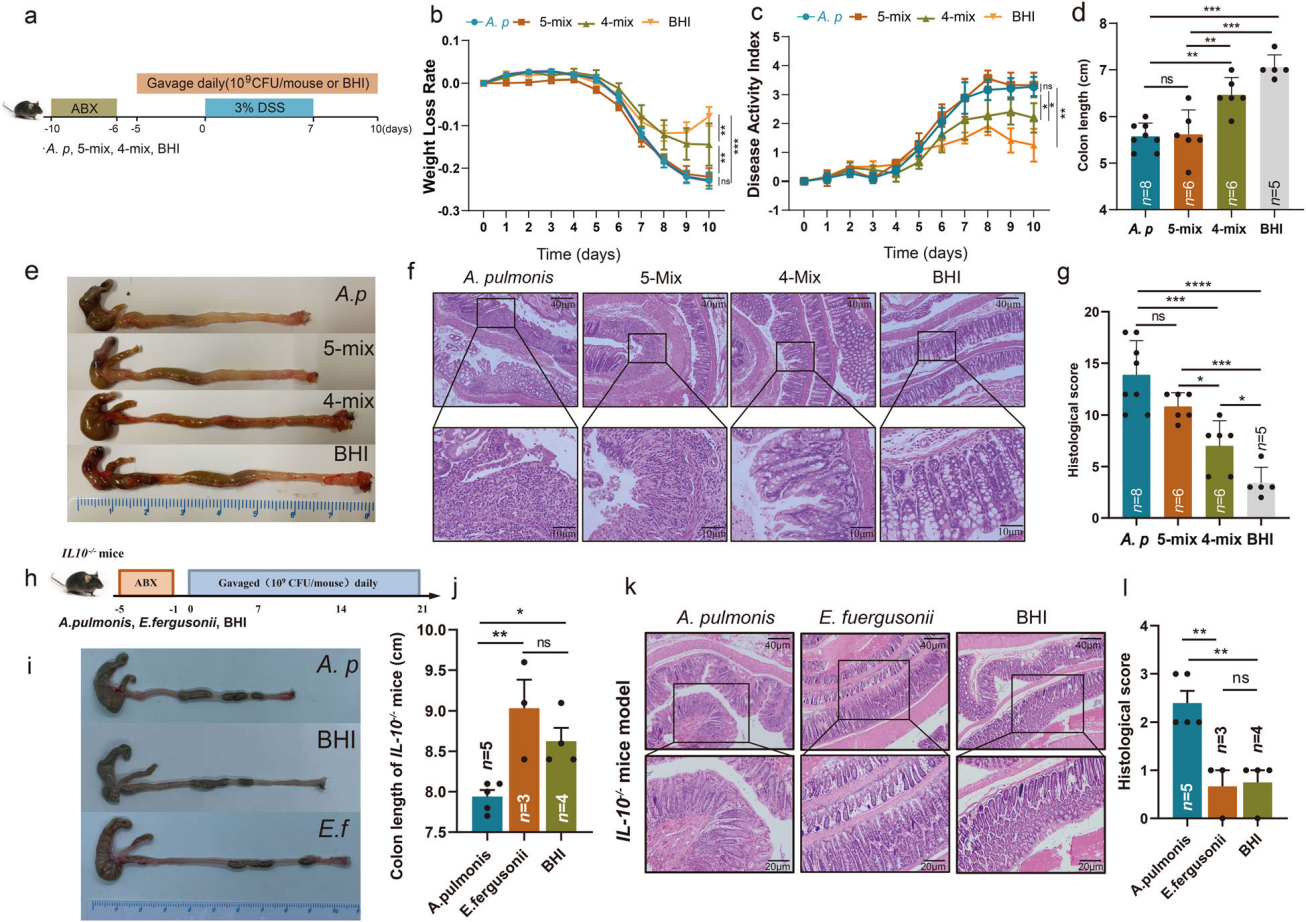
*A. pulmonis* is sufficient to elicit a strong inflammatory response in mice. **a** SPF C57BL/6 mice were treated with an antibiotic cocktail for 4 days. One day post antibiotics, the mice were daily orally colonized with bacterial cocktail (*A. pulmonis*, 5-Mix or residual 4-Mix, 10^9^ CFU/mouse/dose) or BHI until being euthanized. After 5 days bacterial colonization, the mice were exposed to 3% DSS for 7 days, followed by regular water for 3 days. **b** and **c** Changes of body weight (**b**) and disease activity index (DAI) (**c**) after 3% DSS administration. **d–g** Colon length (**d**), representative colons (**e**), representative colonic histological images (Scale bar = 40 μm and 10 μm) (**f**), colonic histological score (**g**) in mice treated with *A. pulmonis*, 5-mix bacterial cocktail, 4-mix bacterial cocktail and BHI. **h** SPF *Il10*^*−/−*^ mice were treated with antibiotics cocktail for 4 days. One day post antibiotics, the mice were daily orally colonized with bacterial cocktail (*A. pulmonis* or *E. fergusonii*, 10^9^ CFU/mouse/dose) or BHI for 3 weeks. **i-l** Colon length (**i**), representative colons (**j**), representative colonic histological images (Scale bar = 40 μm and 20 μm) (**k**), colonic histological score (**l**) from *Il10*^*−/−*^ mice treated with *A. pulmonis*, *E. fergusonii* and BHI. Results are shown as the mean± SEM. Each dot indicates an individual mouse. The statistical significance values are denoted as: *p < 0.05, **p < 0.01, *** *p* < 0.001, ****p < 0.0001. One-way ANOVA following Tukey’s multiple comparison test (**d**, **g, j,** and **l**); two-way ANOVA following Tukey’s multiple comparison test (**b** and **c**)

To verify whether colonization of *A. pulmonis* alone was sufficient to exacerbate colitis, we compared the efficacy of *A. pulmonis* versus the remaining 4 strains (4-mix) from the 5-mix and 5-mix bacteria using the DSS-induced colitis model (Fig. 4a). *A. pulmonis*-colonized mice exhibited comparable weight loss and shortening colons to the mice treated with 5-mix, while the weight loss (Fig. 4b) and shortening colons were moderate in 4-mix-treated mice (Fig. 4d and e). DAI scores in *A. pulmonis* group and 5-mix group were comparable following DSS exposure, which were both significantly worse than those of the 4-mix group (Fig. 4c). Further, histological analysis revealed that both *A. pulmonis*- and 5-mix-colonized mice displayed severe loss of crypt architecture and extensive inflammation (Fig. 4f and g). In contrast, 4-mix-colonized mice had reduced intestinal tissue damage and inflammation. Consistently, fecal presence of *A. pulmonis* in *A. pulmonis*- or 5-mix-colonized mice were significantly higher than other groups (Fig. S9c). To further verify the pro-inflammatory role of *A. pulmonis*, we colonized SPF *Il10*^*-/-*^mice with *A. pulmonis* (10^9^ CFU/mouse) daily after treatment of a broad spectrum antibiotics cocktail (Fig. 4h). Consistent with DSS-induced colitis, *Il10*^*−/−*^ mice colonized with *A. pulmonis* exhibited a significant shortened colon (Fig. 4i and j) and developed more severe colitis after 3-week colonization compared to mice colonized with *E. fergusonii* or BHI (Fig. 4k and l). In line with the histopathologic changes, *A. pulmonis* induced higher colonic gene expression of pro-inflammatory cytokines, such as IL-1β, TNF-α and IL-6 in *Il10*^*−/−*^ mice (Fig. S9d). In the *Il10*^*−/−*^ spontaneous colitis model, *A. pulmonis* colonization significantly downregulated gene expression of several intestinal barrier molecules including *Muc2*, *ZO-1*, and *Occludin* (Fig. S9e). These data suggested that *A. pulmonis* was sufficient to directly exacerbate colitis in both mouse models.

In order to explore pro-inflammatory response of *A. pulmonis*, we co-cultured *A. pulmonis* with murine macrophages cell line RAW264.7, rat intestinal epithelial cell line IEC6, and mouse pre-adipocytes cell line 3T3-L1, and the expression of pro-inflammatory cytokines and chemokines were assessed by RT-PCR. In addition, the accumulation of IL-6, TNF-α, IL-1β, and MCP-1 mRNA were also significantly increased in cells exposed to *A. pulmonis* compared to controls (Fig. S9f-h). Taken together, these results indicated that *A. pulmonis* could directly induce an inflammatory response to mediate colitis development.

### Bacterial translocation into mAT is associated with colitis

**Fig. 5:**
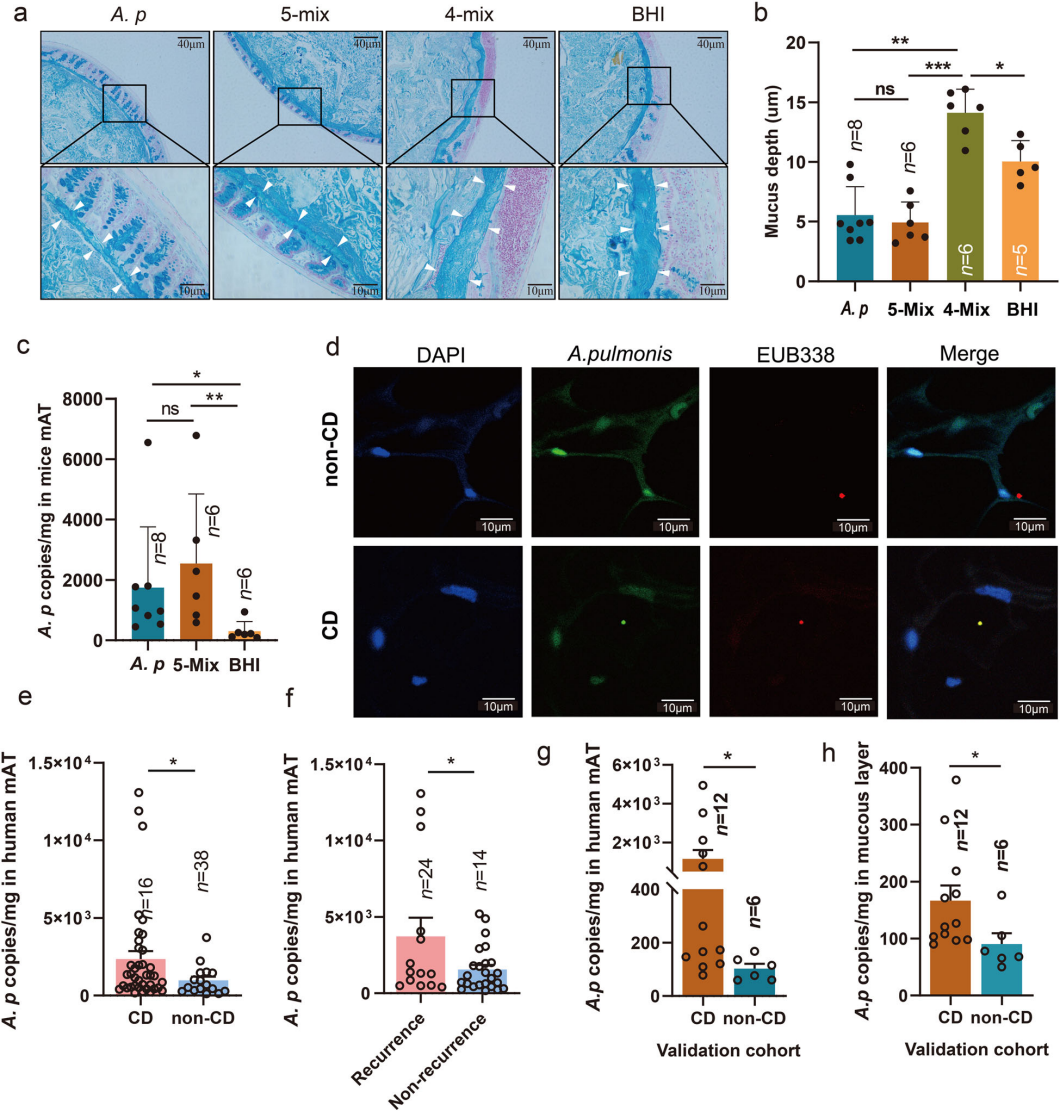
Bacterial translocation into mesenteric adipose tissue is associated with development of colitis. **a** Representative images of mucous depth in the colonic section stained by alcian blue (Scale bar = 40 μm and 10 μm) from mice colonized with bacterial cocktail (*A. pulmonis*, 5-Mix or residual 4-Mix, 10^9^ CFU/mouse/dose) or BHI in DSS-colitis model. **b** Mucous depth from mice treated with *A. pulmonis*, 5-Mix, 4-Mix, or BHI in the DSS-colitis model. **c** The quantity of *A. pulmonis* in mesenteric tissue from mice was assessed by qPCR in the DSS-colitis model. Results are shown as the mean± SEM. **d** Detection of *A. pulmonis* in the human mesenteric tissue (mAT) from CD patients and non-CD controls by fluorescence in situ hybridization (FISH). Mesenteric tissue sections were stained with DAPI (blue), *A. pulmonis*-specific probes (green dot) and EUB338 (red dot). (Scale bar = 10 μm). **e** The quantity of *A. pulmonis* in human mAT from CD and non-CD controls was assessed by qPCR. **f** The quantity of *A. pulmonis* in human mAT from CD patients with and without endoscopic recurrence was assessed by qPCR. **g** and **h** The quantity of *A. pulmonis* in human mAT (**g**) or mucous layer (**h**) from a new validation cohort. Each dot indicates an individual mouse. The statistical significance values are denoted as *p < 0.05, **p < 0.01, ***p < 0.001. One-way ANOVA following Tukey’s multiple comparison test (**b** and **c**); two-tailed Student’s *t*-test (**e–h**)

CD is characterized by increased gut permeability, which could lead to bacterial or bacterial components translocation into the extra-intestinal compartment, such as mAT. The mucous layer is part of the intestinal barrier controlling bacterial translocation. To explore the impact of mAT-associated bacteria on the intestinal barrier, we evaluated various molecules associated with the intestinal barrier. Although expression of different tight junctional molecules was relatively stable, the mRNA level of *Muc2* was significantly downregulated in mice colonized with *A. pulmonis* without DSS intervention (Fig. S10a-d), suggesting an association between *A. pulmonis* and mucous impairment. Consistently, the mucous layer thickness from *A. pulmonis*-colonized mice was significantly decreased compared to those from control groups in DSS-induced colitis (Fig. 5a and b). Furthermore, an increased presence of *A. pulmonis* was observed in extra-intestinal mAT in the DSS colitis model (Fig. 5c). These results suggest that bacterial translocation was associated with impairment of intestinal mucous layer by mesenteric bacteria from CD patients.

To further confirm the bacterial translocation into mAT in CD patients, we performed fluorescence in situ hybridization (FISH) using *A. pulmonis*-specific probes and general eubacterial probes under sterile conditions visualize and quantify *A. pulmonis* in human mAT. This analysis showed increased presence of *A. pulmonis* in the mAT from CD patients (Fig. 5d and Fig. S11), but decreased in those from non-CD control (Fig. S11). Furthermore, we quantified *A. pulmonis* 16S rRNA gene in human mAT using quantitative RT-PCR and found that *A. pulmonis* were significantly higher in mAT from CD patients compared with those in non-CD controls (Fig. 5e), whereas the total bacterial DNA was also higher in mAT from CD patients than those from non-CD controls (*p* = 0.0591) (Fig. S12c). As for the patients with endoscopic recurrence, the level of *A. pulmonis* in mAT from patients with endoscopic recurrence was significantly higher than those without endoscopic recurrence (Fig. 5f).

Although the colitogenic role of *A. pulmonis* from mAT has been evaluated, their role in mesenteric translocation is limited. To assess the potential source of mAT associated microbiota, we retrieved fecal 16S rRNA gene sequencing data from the RISK cohort [21]. Similar to the result in our cohort, the abundance of Alcaligenaceae, Brucellaceae, Pseudomonadaceae, and Hyphomicrobiaceae were significantly upregulated in feces from CD than those from non-CD controls (Fig. S12a). More specifically, we found that the abundance of *Achromobacter* spp. from Alcaligenaceae was also significantly higher in feces from CD than those from non-CD controls (Fig. S12b). To further evaluate the mesenteric translocation of *A. pulmonis*, a new clinical cohort including mATs and corresponding mucosal tissue from 12 CD patients, as well as the mesenteric and mucous tissue from 6 non-CD controls, were recruited for validation analysis. Colonization of *A. pulmonis* in mAT by qPCR detection was significantly higher than those from non-CD controls (Fig. 5g). Consistently, the signal of total bacterial DNA and *A. pulmonis* were both significantly higher in the corresponding mucosal layer from CD patients than those from non-CD controls (Fig. 5h and Fig. S12d). These data suggested that high colonization of *A. pulmonis* in mAT was associated with the bacterial translocation from the gut microenvironment.

## Discussion

The present study characterizes and elucidates the deleterious role of mAT resident microbiota in CD development using a multi-omics approach and animal models. We showed that translocated *A. pulmonis* into mAT exhibited an ability to induce experimental colitis in both DSS and *Il10*^*−/−*^ mouse models. Our findings finally highlighted the role of the intestinal barrier in preventing bacterial translocation to mAT and colitis development. Together, our study revealed that bacteria-mediated alteration of intestinal barrier function favors microbial translocating into mAT and formation of a unique pro-inflammatory bacterial community mediating the colitis development.

A number of studies have reported an association between the presence of bacterial DNA in AT and development of inflammation [22]. Alteration of microbial composition has also been found to be associated with obesity, systemic inflammation, and liver fibrosis [23–25]. A study from a CD cohort found a microbial signature in mAT, characterized by an increased abundance of Proteobacteria [14]. But the small sample size of this cohort and lack of functional studies limited the biological impact of the study. Recently, Ha et al. demonstrated that translocation of viable gut microbiota in mAT drove the formation of creeping fat [15]. Although the role of microbiome in the formation of the creeping fat had been revealed, the clinical relevance of creeping fat in CD patients is still unknown. The previous clinical study had demonstrated the protective outcome for removing mesentery in ileocolic resection for CD [5], especially the improvement of post-operative recurrence, raising a controversy for the role of mAT-associated microbiota in CD development. Hence, our current study focused on the role of mAT-associated microbiota in CD development. Our prospective cohort of 64 patients demonstrated the presence of dysbiosis in mAT of CD patients, and more importantly, highlighted the functional role of Proteobacterium phylum in disease phenotype. Consistent with the role of ileal mucosa-associated Proteobacteria in post-operative endoscopic recurrence of CD [26], our study demonstrated that mAT resident pathogens were also associated with the poor prognosis of post-operative CD patients. In addition to the microbiotic sequencing of mAT, we had intended to isolate various microbes from CD mesenteric tissue. Although bacteria present in mAT from CD patients are not overlapping between studies [15], multiple microbes from Proteobacteria were consistently enriched in mAT from CD patients, while some bacteria such as *Streptococcus* and *Lactobacillus* were enriched in mAT from those non-CD controls in both studies [15]. Despite the evidence of bacteria staining in AT [27], to our knowledge, no study had identified colitogenic isolates from mAT. These findings together with our study have a strong physiological significance that mAT-associated bacteria represent an important virulence trait implicated in CD development.

Although multi-omics analysis of gut microbial ecosystem has been performed using feces or intestinal tissue of IBD patients [28, 29], system-level understanding of the etiology of mAT of CD patients has not been completed. Our study found a tight coupling of metabolome and microbiota in mAT, indicating a perturbation of metabolite resulting from altered microbiota. To better characterize the mesenteric microenvironment, we studied the transcriptomic profile. Alteration of the mesenteric transcriptome was associated with aberrant extracellular architecture and intercellular adhesion, similar to the result of single-cell sequencing from creeping fat which was recently demonstrated by Ha et al. [15]. Interestingly, the differences in microbiome were relatively modest compared to transcriptome and metabolome in mAT from CD patients compared to those from non-CD controls, which suggests that microbial functional features but not phylogeny contribute most to these differences. Notably, our study further suggested that mesenteric-associated microbiota may drive the alteration of host transcripts, increasing the migration of stromal cells and therefore resulting in the formation of creeping fat.

The previous study had shown that various microbes isolated from CD patients could mediate intestinal inflammation in animal models [15, 30, 31]. Here, we identified a group of bacteria, particularly, *A. pulmonis* that possessed colitogenic capacity in vitro and in vivo. This bacterium has been previously identified as an opportunistic pathogen [32]. Obata et al. have previously identified the presence of *Achromobacter* spp*.* in lymphoid follicles, Peyer’s patches, and mesenteric lymph nodes of healthy mammalian hosts [32]. Further study also found the presence of bacterial DNA of Alcaligenaceae in the liver and spleen [33]. However, the function of *Achromobacter* spp. in vivo remains unclear. Interestingly, our 16S rRNA gene sequencing analysis from a publicly available database indicated that *Achromobacter* spp. was enriched in the feces from CD patients compared to non-CD controls [21]. As the previous study has demonstrated the similar diversity between mucous tissue and mAT [15], our two clinical cohorts also found that a higher level of *Achromobacter* in mAT from CD patients might translocate from the intestinal lumen. Besides this, the previous study also showed that *Clostridium innocuum* isolated from mAT of CD patients could translocate to mAT in mice experiment and promote adipose tissue expansion [15]. Notably, the enriched abundance of this bacterium in mAT was also associated with post-operative recurrence. The higher prevalence of mAT-associated *Achromobacter* spp. in patients with CD, in conjunction with the ability of *A. pulmonis* to promote colitis in mice, point to a potential pathogenetic role of this bacterium in CD. More importantly, the presence of pathogens in mAT exhibited an excellent performance in the prediction of CD prognosis, suggesting the necessity of inclusive mesenteric resection in CD patients.

CD is characterized by altered epithelial permeability and increased bacterial translocation [34]. Interestingly, bacteria are frequently detected in both mAT and mesenteric lymph nodes from CD patients [35, 36]. Various mouse models of colitis, including mice deficient in *Muc2*, *Tlr5*, *Il10*, or DSS-induced colitis model, had bacteria penetrating into the otherwise impenetrable inner colonic mucous layer [37]. Interestingly, we observed that colonization of mice with *A. pulmonis* led to impaired intestinal mucous layer and thus translocated into mAT and exacerbate colitis both in the DSS-induced colitis model and in the *Il10*^*−/−*^ mouse model. This suggests that bacteria can translocate into mAT via impairment of the mucous layer in pathological conditions, which is supported by recent studies [38, 39]. Approximately 1% of intestinal bacterial species possessed the ability to produce the requisite extracellular enzymes to degrade the colonic mucous layer [40]. Specific mucolytic enzymes, such as glycosidase, sulfatase, and sialidase enzymes, were found in bacteria spanning several genera [41–45]. Strikingly, Chen et al. previously isolated a novel species of *Achromobacter* that could produce β-glucosidase [46, 47], implicating the potential mechanism of *A. pulmonis* in mucous impairment. Genomic characterization of our *A. pulmonis* isolate will further help understand the mucolytic capacity of this bacterium.

Our present study highlighted the colitogenic role of mAT-associated bacteria in colitis development. However, some limitations still remained in our present study. Although there is distinct differences in multi-omics measurement between mAT from CD and non-CD, the inclusion of non-hyperplastic mAT from patients with CD would be needed to definitely support our conclusion. Furthermore, more evidences were needed to evaluate the systemic bacterial dissemination and those CD patients treated with immunotherapy. Our data suggest the existence of pro-inflammatory pathobionts in mAT. While the previous study suggested a beneficial role of creeping fat in human CD, it is likely that persistent inflammatory stimuli could lead to adipogenesis and fibrosis. The role of *A. pulmonis* in adipogenesis and fibrosis warrants further investigation. In addition, the localized bacterial dissemination and inflammation by adipogenesis appeared to be associated with a persistent bacterial stimulation to its surrounding intestine. Further experiments are encouraged to clarify the underlying mechanism.

## Conclusions

This study provides evidences of a dysregulated microbiota–host interaction in mAT which is associated with colitis development in CD patients. Microbiota, especially *A. pulmonis*, from mAT can translocate to mAT and promote exacerbation of colitis in mice. Understanding bacterial contribution to CD development can lead to novel treatments based on targeting microbial-derived activity.

## Supplementary Information

**Additional file 1.**

## Data Availability

Data are available on reasonable request. All data relevant to the study are included in the article or uploaded as supplementary information.
