# Supplementary material for: Microbiota in mesenteric adipose tissue from Crohn’s disease promote colitis in mice
Source: Microbiome. 2021 Nov 23;9:228. doi: 10.1186/s40168-021-01178-8 (PMC8609859; doi:10.1186/s40168-021-01178-8)
Supplement: Supplementary file 2 — Additional file 1. [file 40168_2021_1178_MOESM2_ESM.docx]

**Supplementary methods**

**Study design and sample collection**

Forty-eight patients with CD undergoing ileocolectomy surgery were recruited at the Sixth Affiliated Hospital of Sun Yat-Sen University. Sixteen patients diagnosed with colorectal cancer based on non-specific symptoms, endoscopic and histopathologic finding of CD, were classified as non-CD controls. All patients underwent the collection of anthropometric and routine clinical phenotyping. Individuals were excluded if they were unable to or did not consent to provide tissue, had taken antibiotics medication within 2 weeks, were contraindicated to surgery, had an acute gastrointestinal infection or perforation, were pregnant, had a known bleeding disorder, were diagnosed with end-stage malignancy. After surgery, a colonoscopy was performed to assess the endoscopic recurrence according to the Rutgeerts score. Post-operative recurrence was defined by a Rutgeerts score of ≥i2.

Mesenteric fat samples were collected before opening the intestine to prevent spill-over contamination during surgery. Mesenteric tissue was excised from the hyperplastic mesenterium close to the terminal ileum and immediately put in sterile collection tube and subsequently put on ice. Those mAT from non-CD controls were resected from the terminal ileum, the same location where CD mAT was collected from. Mesenteric tissue from each subject was divided into 4-5 pieces in an anaerobic chamber. Four of them were respectively subjected for 16S rRNA gene sequencing, metabolic analysis, RNA sequencing and microbial isolation.

**High throughput 16S rRNA amplicon sequencing and analysis**

Genomic DNA was extracted by using a modified protocol of the DNeasy Blood & Tissue Kit (QIAGEN, Germantown, MD). For 16S rRNA gene sequencing, the V4 variable region was amplified by PCR with dual barcoded primers as previously described^1^. The amplicons were purified using AMPure XP (Beckman Coulter) and quantified using the Quant-iT PicoGreen ds DNA Assay Kit (Thermo Fisher Scientific). The pooled amplicons were further qualified and quantified by a Bioanalyzer 2100 with the High Sensitivity DNA Kit (Agilent) and the KAPA Library Quantification Kit for Illumina (Kapa Biosystems). The denatured amplicons were mixed with 20% PhiX Control v.3 and sequenced on a HiSeq (Illumina, 2 × 250-bp paired-end reads). An open reference-based 16S rRNA gene amplicon sequence variants (ASVs) pickup strategy was performed to quantify the mAT samples. The Quantitative Insights into Microbial Ecology 2 (QIIME2) platform^2^ was employed with recommended parameters to demultiplex and quality filter. Then, the 16S V4 sequences with similarity 100% were clustered into ASVs. Taxon of an ASV was assigned by UCLUST^3^ algorithm based on the GreenGene database^4^. The relative abundance of each ASV was determined as a proportion of the sum of sequences for each sample and a random of 10,000 tags were selected for each sample to avoid depth bias of sampling.

**Host RNA sequencing and analysis**

All mAT from our cohort were recruited for RNA sequencing analysis, whereas 3 of these samples were failed to extract RNA (2 in CD cohort and 1 in non-CD cohort). Total RNA was extracted from mesenteric tissues using Trizol® Reagent (Life Technologies, Carlsbad, CA, USA) and precipitated with isopropanol. RNA integrity was evaluated both by agarose gel electrophoresis and BioAnalyzer analysis. The RNA-seq libraries (non-strand-specific, paired end) were prepared using TruSeq RNA Sample Prep kit (Illumina, San Diego, CA), and the libraries were successfully constructed from mesenteric samples. To construct the RNA-seq libraries, at least 100 ng of the total RNA were transcribed into cDNA, and the quantity of the cDNA libraries were assessed by Nanodrop instrument (Thermo-Fisher). Then, we used a covaries S220 system (Covaris, Woburn, MA) to generate sheared cDNA, and quantify the cDNAs by Nanodrop again to calculate the appropriate amount of cDNA for library construction. Sample-specific barcodes were added to each cDNA library, and then pooled and sequenced on HiSeq platform (illumine, San Diego, CA) with a 2×100bp HiSeq run. Differential expression analysis of two groups (two biological replicates per condition) was performed using the DESeq2 R package (1.16.1). DESeq2 provide statistical routines for determining differential expression in digital gene expression data using a model based on the negative binomial distribution^5^. The resulting p-values were adjusted using the Benjamini and Hochberg’s approach for controlling the false discovery rate. Genes with an adjusted p-value <0.05 found by DESeq2 were assigned as differentially expressed^6^.

**Untargeted metabolomics of mesenteric samples**

Untargeted measurement of metabolites in mAT was performed using liquid chromatography-triple quadrupole mass spectrometry (LC−MS/MS). LC-MS/MS analyses were performed using an UHPLC system (1290, Agilent Technologies) with a UPLC HSS T3 column (2.1 mm × 100 mm, 1.8 μm) coupled to Q Exactive mass spectrometer (Orbitrap MS, Thermo). The mobile phase A was 0.1% formic acid in water for positive mode, and 5 mmol/L ammonium acetate in water for negative mode, and the mobile phase B was acetonitrile. The elution gradient was set as follows: 0~1.0 min, 1% B; 1.0~8.0 min, 1%~99% B; 8.0~10.0 min, 99% B; 10.0~10.1 min, 99%~1% B; 10.1~12 min, 1% B. The flow rate was 0.5 mL/min. The injected volume was 3 μL. The QE mass spectrometer was used for its ability to acquire MS/MS spectra on information-dependent acquisition (IDA) mode in the control of the acquisition software (Xcalibur 4.0.27, Thermo). In this mode, the acquisition software continuously evaluates the full scan MS spectrum. The ESI source conditions were set as following: sheath gas flow rate as 45 Arb, Aux gas flow rate as 15Arb, capillary temperature 400 ℃, full MS resolution as 70000, MS/MS resolution as 17500, collision energy as 20/40/60 in NCE mode, spray Voltage as 4.0 kV (positive) or -3.6 kV (negative), respectively. In this study, mAT-associated metabolites were left after relative standard deviation de-noising. Then, the missing values were filled up by half of the minimum value. Also, total ion current normalization method was employed in this data analysis. The final dataset containing the information of peak number, sample name and normalized peak area was imported to SIMCA15.0.2 software package (Sartorius Stedim Data Analytics AB, Umea, Sweden) for multivariate analysis. Data was scaled and logarithmic transformed to minimize the impact of both noise and high variance of the variables. Supervised orthogonal projections to latent structures discriminate analysis (OPLS-DA) was applied to visualize group separation and find significantly changed metabolites. Then, a 7-fold cross validation was performed to calculate the value of R2 and Q2. R2 indicates how well the variation of a variable is explained and Q2 means how well a variable could be predicted. To check the robustness and predictive ability of the OPLS-DA model, a 200 times permutations was further conducted. Afterward, the R2 and Q2 intercept values were obtained. Here, the intercept value of Q2 represents the robustness of the model, the risk of overfitting and the reliability of the model, which will be the smaller the better. The value of variable importance in the projection (VIP) of the first principal component in OPLS-DA analysis was obtained. It summarizes the contribution of each variable to the model^7^. The metabolites with VIP>1 and p<0.05 (student t test) were considered as significantly changed metabolites.

**Effect size analysis**

In this study, we performed the “effect size” analysis strategy to determine whether the omic datasets can affect each other. To assess the proportion of variance of an omic dataset that be explained by another omic dataset, the adoins function of the R package vegan^8^ was used to estimate the “one-to-all” effect size (R2) between each single variable of the secondary omic to the whole original omic dataset. Only variable with p< 0.05 was considered to exhibit significant effect on the original omic dataset.

**Multivariate analysis**

Multivariate statistical analysis were applied to discriminate CD patients from non-CD individuals. Principle component analysis (PCA) was performed on the mesenteric microbiome, metabolomes and transcriptome using the ade4 package in R platform^9^.

**Integrated analysis of multi-omics**

The relationships of all variables among omic datasets were established using correlation network analysis as follow: 1) Spearman correlation coefficient between mesenteric metabolome, immune-associated transcripts and mesenteric microbiome was calculated. The adjusted p value was determined and the threshold of 0.05 was accepted; 2) to identify the key role of microbiome in the network, the entire network was parsed, the number of connections of every datasets was calculated. The correlation relationship of omics variables were calculated on both CD patients and non-CD control, respectively; 3) the network diagrams were visualized by Cytoscape^10^ using circular layout.

**Random forest model**

The predictability of the mesenteric microbiome to disease stratification was estimated using random forest package based on the microbiotic profiles. In order to produce a strong classifier, Random forests model tries to grow multiple decision (CART) trees with different samples and different initial variables. Each tree gives a classification. Lists of taxa ranked by Random Forests in order of feature importance were determined over 100 iterations. The number of marker taxa were identified using 10-fold cross-validation implemented with the rfcv() function in the R package “randomForest” with five repeats^11^. The minimum cross-validation error was obtained when using 16 important families. The accuracy of the model was examined using a ROC curve.

**LASSO logistic regression model**

In order to identify microbiotic markers that can distinguish CD samples from samples of non-CD control, LASSO logistic regression model^12^ was constructed by glmnet package in R. Models were validated by 10-fold stratified cross-validation testing. Lambda.min returned the value of λ that gives minimum mean cross-validated error. Based on the value of Lambda.min, the most relevant ASVs were obtained in the detection of CD. The accuracy of the model was examined using a ROC curve.

**Mantel test**

Quantifications of covariation between microbiome and transcriptome were done using Mantel test as previous described^13^. To quantify cross-sectional (“inter-individual”) covariation, we first produced an average profile for each subject by taking the feature-wise mean over all samples from the subject. Subject-subject dissimilarity matrices were then generated and compared using the mantel.rtest function in the R package ape4. To quantify longitudinal covariation, we first generated the complete sample–sample dissimilarity matrix, but only calculate the Mantel test statistic (the Pearson correlation between distances) from distances between samples from the same subject. Significance in this case was assessed using a permutation test with permutations limited within-subject.

**Isolation and identification of mesenteric bacteria**

For the isolation of mesenteric bacteria, fresh resected tissues were chopped and homogenized as previously described. After series dilutions, the supernatant was spread onto brain heart infusion (BHI) agar plate, BHI supplemented agar plate, tryptose soya agar (TSA) plate or MacConkey agar plate, and incubated overnight under aerobic or anaerobic conditions for 72 hours (80% N_2_, 10%H_2_, 10%CO_2_), respectively. Bacterial DNA was extracted from each single colony and the identity of individual isolates was verified by Sanger sequencing of the V1-V9 regions of 16S rRNA gene. The following primer set was used for amplifications: 27F 5'-AGAGTTTGATCCTGGCTCAG-3' and 1492R 5'-GGTTACCTTGTTACGACTT-3'. 16S rRNA sequencing was performed using the Illumina MiSeq platform.

**Mice experiment**

Six- to eight-week-old male C57BL/6 mice were housed with a 12 hours’ light–dark cycle in specific pathogen-free (SPF) facilities at the South China Agricultural University. SPF mice were fed a sterilized laboratory rodent diet 5L0D (LabDiet). Animals were handled in accordance with the protocols approved by the Institutional Animal Care and Use Committee (IACUC) at the Sun Yat-Sen University. All SPF male mice were treated with broad-spectrum antibiotic cocktail (ampicillin 0.2g/L, metronidazole 0.2g/L, Neomycin 0.2g/L, vancomycin 0.1g/L) in the drinking water for 4 days. For mesenteric pathobiont colonization experiments, mice were gavage with pathobionts (1×109 CFU/dose) everyday. At day7, mice received 3.0% DSS for 7 days followed by four days regular drinking. The animals were monitored for weight loss (0, none; 1, 1%–5%; 2, 5%–10%; 3, 10%–20%; 4, > 20%), stool consistency (0, normal stool; 2, loose stool; 4, diarrhea), and hemoccult (0, normal; 2, hemoccult positive; 4, gross blood) during the course of experiments, and these parameters were used to compute the disease activity index (DAI)^14^. Mice were sacrificed on day 16 and their colon tissues were fixed in 4% paraformaldehyde for HE staining or modified Carnoy’s fixative for Alcian staining. Histological scores were assigned in a blind manner by a trained pathologist evaluating the following set of variables: severity of inflammation (0, none; 1, low density confined to mucosa; 2, moderate or higher density in mucosa and/or low to moderate density in mucosa and submucosa; 3, high density in submucosa and/or extension to muscularis; 4, high density with frequent transmural extension), and extent of epithelial/crypt damage (0, none; 1, basal 1/3; 2, basal 2/3; 3, crypt loss; 4, crypt and surface epithelial destruction). Each variable was multiplied by a factor reflecting the percentage of the colon involved (1, 0%–25%; 2, 26%–50%; 3, 51%–75%; 4, 76%–100%). An overall score was obtained by summing the scores assigned to each variable^15^. For experiment using *Il10* deficient (*Il10^-/-^* ) model, mice were gavaged with pathobionts (1×10^9^ CFU/dose) for 3 weeks. Histologic analysis was performed in a blinded fashion, with regions graded from 0 to 4 (0 = normal, 1 = mild, 2 = moderate, 3 = marked, and 4 = severe) based on the degree of lamina propria and submucosal mononuclear cellular infiltration, crypt hyperplasia, goblet cell depletion and architectural distortion. A score of 0 means no signs of inflammation; a score of 1 means mild leukocytic infiltration in lamina propria with mild epithelial hyperplasia and goblet cells depletion; a score of 2 represents moderate inflammation involving submucosa, occasional crypt abscesses, moderate crypt hyperplasia and goblet cells depletion; a score of 3 means marked inflammation involving the submucosa and muscular layers, and marked crypt hyperplasia with few goblet cells; a score of 4 represents severe/maximal inflammation involving all characteristics in “3” plus multiple crypt abscesses, mucosal ulceration and transmural inflammation. For the experiment of butyrate intervention, sodium butyrate was administered in drinking water (3g/L) for mice every day.

**Cell culture**

The murine cell lines RAW264.7, 3T3-L1 and rat cell line IEC6 cells were purchased from American Type Culture Collection (ATCC). All of the cells were cultured at 37 °C in Dulbecco’s modified Eagle's medium (DMEM; Gibco, Thermo Fisher Scientific, St Peters, MO, USA) supplemented with 10% fetal bovine serum (FBS; Gibco, Thermo Fisher Scientific, St Peters, MO, USA) in a 5% CO_2_ atmosphere.

**In vitro stimulation by bacteria**

Murine macrophage cell lines RAW264.7 and isolated mesenteric bacteria were co-cultured with a multiplicity of infection (MOI) of 10 at 37 °C, 5% CO_2_ for 1h. Gentamycin (100 uL/ml) and Kanamycin (100 uL/ml) were then added and the cells were further cultured for 1h. For bacteria-cells co-culture experiments, pre-adipocytes 3T3L-1 or epithelial cells IEC6 were co-cultured with *A. pulmonis* (MOI=20) at 37 °C, 5% CO_2_ for 4 h. Cells treated with phosphate buffer solution (PBS) were used as control. Kanamycin (100 uL/ml) was then added and cells were further cultured for 1h. RNA from infected cells were extracted and cytokines were measured by qPCR. For cytotoxicity analysis, cells were infected with bacteria with a MOI of 20 for 24 h. The release of lactate dehydrogenase (LDH) was evaluated by an LDH Cytotoxicity Assay kit (Beyotime, China).

**RNA extraction and Real-time quantitative PCR analysis**

Colonic tissue samples were harvested, and RNA was extracted using Total RNA Kit (R323-01, Vazyme, China). cDNA was reverse transcribed using a Hiscript@ III RT Super Mix with gDNA wiper ( R323-01, Vazyme, China). Real-time PCR was performed on the Applied Biosystems 7500 Real-time PCR system using SYBR Green Real-time PCR Master Mix (QPK-201, Toyobo, Japan). Primer sequences used in this study was shown in **Supplementary table 6**.

**Fluorescence in situ hybridization (FISH)**

For the visualization of bacteria, Fluorescence in situ hybridization was performed following a modified protocol described previously^16^. All steps were performed under sterile conditions. The probes used in this study was *A. pulmonis* -speicific probe, a genus-specific probe complementary to a 16S rRNA gene region of Achromobacter. All probes were 5’-labelled with digoxigenin, and antidigoxigenin/horseradish peroxidase antibodies were used as secondary antibodies. Paraffin-embedded sections were subjected to deparaffinisation, rehydration and permeabilisation. Deparaffinised sections were sequentially treated with buffer A, B as well as C for 10 min at 37°C to achieve permeabilisation. Hybridization with *A. pulmonis*-speicific probe was performed at 46°C overnight, and afterwards slides were treated with wash buffer for 10 min at 48°C three times, Tris-buffer for 5min at 48°C three times and for 1 min in 1×PBS. After staining with DAPI (1 ug/ml) for 10 min at room temperature, six areas of each section were randomly photographed under a fluorescence microscope.

**Quantification and statistical analysis**

Unless otherwise stated in individual method sections above, all statistical analyses were performed using Prism 8 (GraphPad Software, San Diego, CA). Differences between two groups were evaluated using the Student t test (parametric) or Mann–Whitney U test (non-parametric). For a comparison of more than 3 groups, statistical analysis was performed using one-way ANOVA (parametric) or Kruskal–Wallis test (non-parametric). Differences of adjusted p < 0.05 were considered significant in all statistical analyses. Statistically significant differences are shown with asterisks as follows: *, p < 0.05; **, p < 0.01; ***, p < 0.001 and ****, p < 0.0001; whereas, ns indicates comparisons that are not significant. Numbers of animals (n) used for individual experiments, details of the statistical tests used and pooled values for several biological replicates are indicated in the respective figure legends.

**Supplementary Reference:**

1. Kozich JJ, Westcott SL, Baxter NT, et al. Development of a dual-index sequencing strategy and curation pipeline for analyzing amplicon sequence data on the MiSeq Illumina sequencing platform. Appl Environ Microbiol 2013;79:5112-20.

2. Kuczynski J, Stombaugh J, Walters WA, et al. Using QIIME to analyze 16S rRNA gene sequences from microbial communities. Curr Protoc Microbiol 2012;Chapter 1:Unit 1E.5.

3. Edgar RC. Search and clustering orders of magnitude faster than BLAST. Bioinformatics 2010;26:2460-1.

4. DeSantis TZ, Hugenholtz P, Larsen N, et al. Greengenes, a chimera-checked 16S rRNA gene database and workbench compatible with ARB. Appl Environ Microbiol 2006;72:5069-72.

5. Finn RD, Bateman A, Clements J, et al. Pfam: the protein families database. Nucleic Acids Res 2014;42:D222-30.

6. Mao X, Cai T, Olyarchuk JG, et al. Automated genome annotation and pathway identification using the KEGG Orthology (KO) as a controlled vocabulary. Bioinformatics 2005;21:3787-93.

7. Saccenti E, Hoefsloot HCJ, Smilde AK, et al. Reflections on univariate and multivariate analysis of metabolomics data. Metabolomics 2014;10:361-374.

8. Oksanen J, Kindt R, Legendre P, et al. The Vegan Package. Community ecology package 2007:631-637.

9. Legendre P, Anderson MJ. Distance-based redundancy analysis: Testing multispecies responses in multifactorial ecological experiments. Ecological Monographs 1999;69:1-24.

10. Smoot ME, Ono K, Ruscheinski J, et al. Cytoscape 2.8: new features for data integration and network visualization. Bioinformatics 2011;27:431-2.

11. Zhang J, Zhang N, Liu YX, et al. Root microbiota shift in rice correlates with resident time in the field and developmental stage. Sci China Life Sci 2018;61:613-621.

12. Tibshirani R. Regression Shrinkage and Selection Via the Lasso. Journal of the Royal Statistical Society: Series B (Methodological) 1996;58:267-288.

13. Lloyd-Price J, Arze C, Ananthakrishnan AN, et al. Multi-omics of the gut microbial ecosystem in inflammatory bowel diseases. Nature 2019;569:655-662.

14. Cooper HS, Murthy SN, Shah RS, et al. Clinicopathologic study of dextran sulfate sodium experimental murine colitis. Lab Invest 1993;69:238-49.

15. Klopfleisch R. Multiparametric and semiquantitative scoring systems for the evaluation of mouse model histopathology--a systematic review. BMC Vet Res 2013;9:123.

16. Au - Choi YS, Au - Kim YC, Au - Baek KJ, et al. In Situ Detection of Bacteria within Paraffin-embedded Tissues Using a Digoxin-labeled DNA Probe Targeting 16S rRNA. JoVE 2015:e52836.

**Supplementary Figure**

**
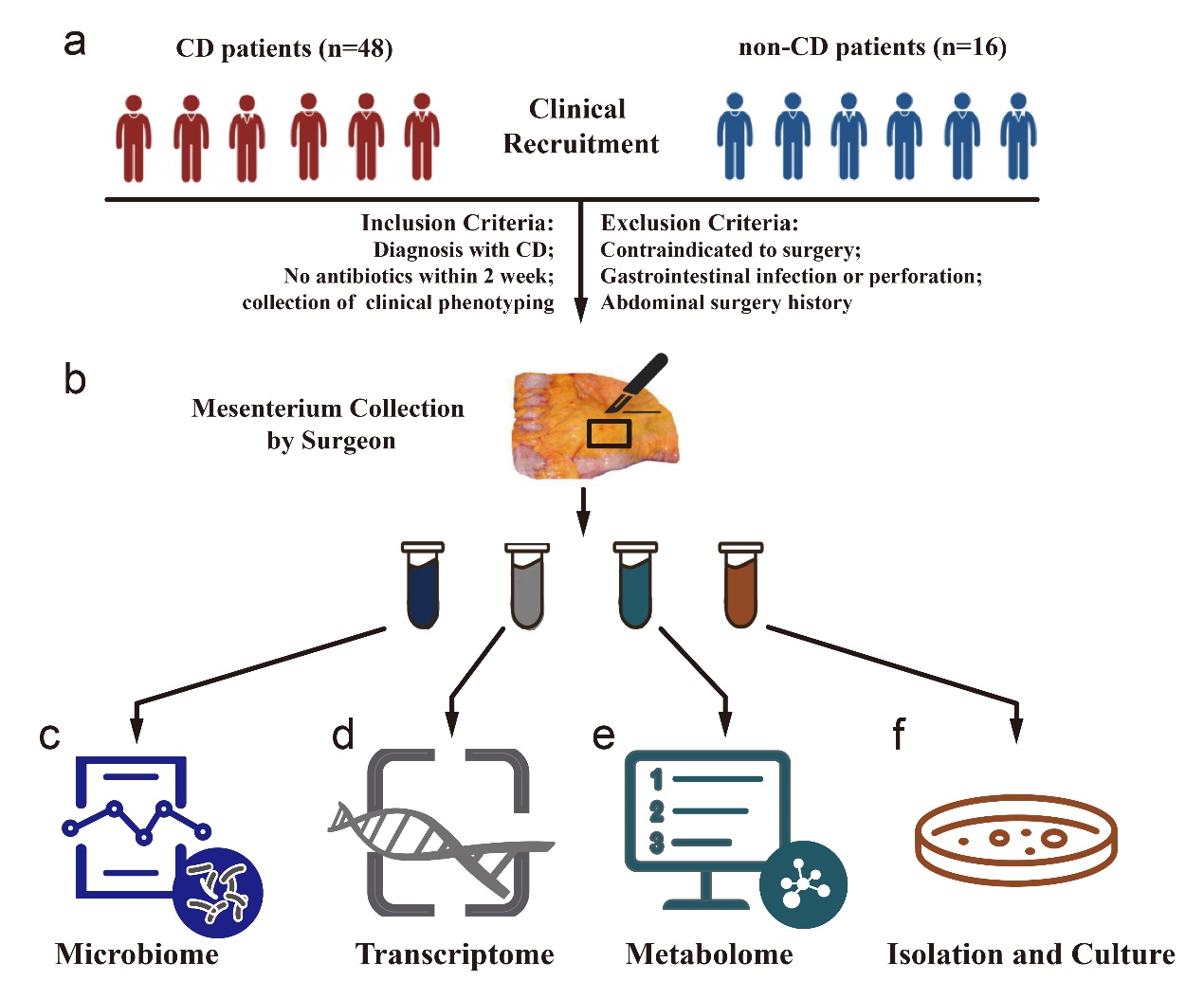
**

**Fig. S1 Overview of the workflow for the multi-omics strategy used in this study.** **(a)** A cohort of 64 patients consisting 48 patients diagnosed with CD and 16 patients diagnosed with non-CD were prospectively recruited into our study. **(b)** Mesenteric adipose tissue (mAT) of terminal ileum were collected by skilled surgeons. **(c-e)** Integration of microbiome (**c**), transcriptome (**d**) and metabolome (**e**) data were subsequently analyzed. **(f)** mAT resident bacteria were isolated under anaerobic and aerobic conditions using different culture media.

**
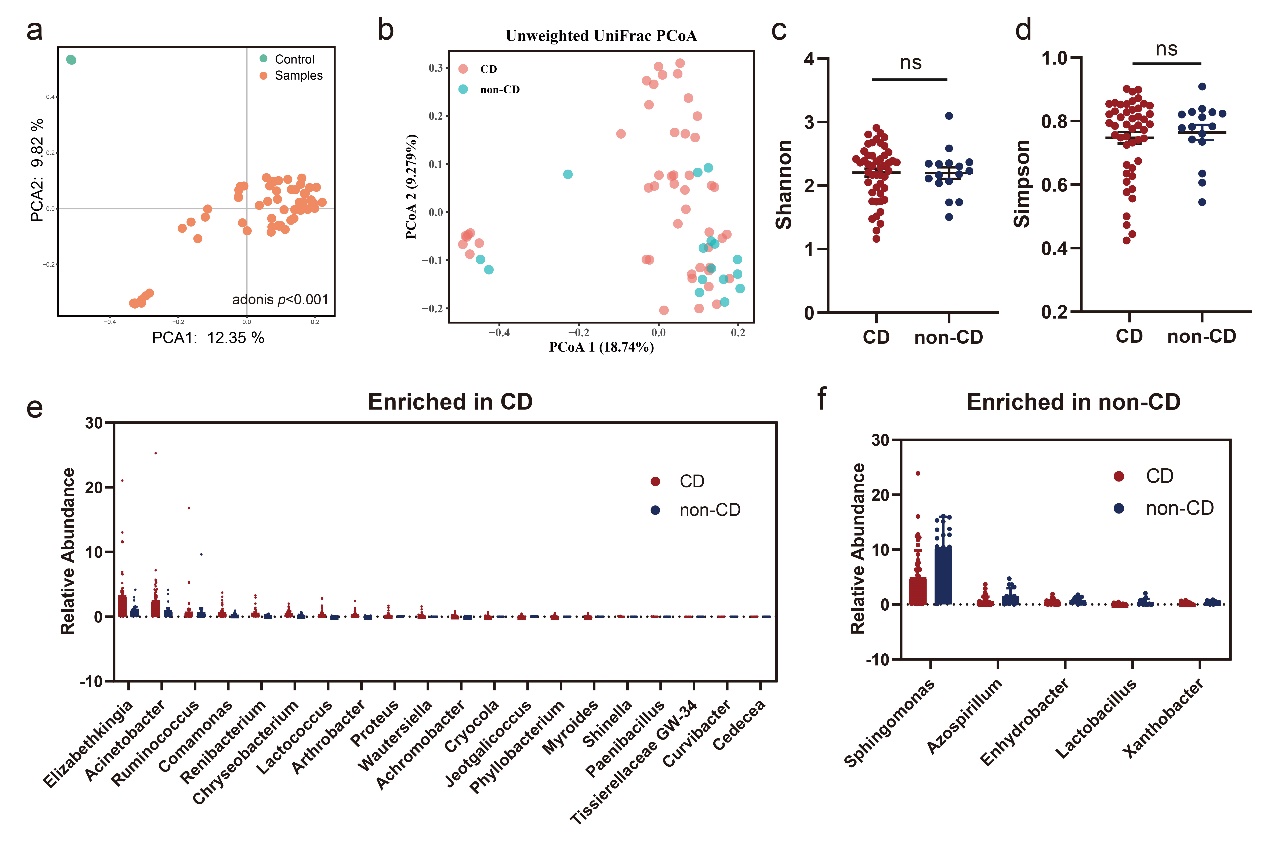
**

**Fig. S2 Differential analysis of microbiome in mAT.** **(a)** principal component analysis (PCA) between mAT samples (n=64) and quality controls (n=4). **(b)** Principal coordinates analysis (PCoA) of microbiome between mAT from CD and non-CD controls. **(c and d)** Alpha-diversity of microbiome between mAT from CD and non-CD controls: Shannon index (**c**) and Simpson index (**d**). **(e)** The microbes significantly enriched in mAT from CD patients. **(f)** The microbes significantly enriched in mAT from non-CD controls.


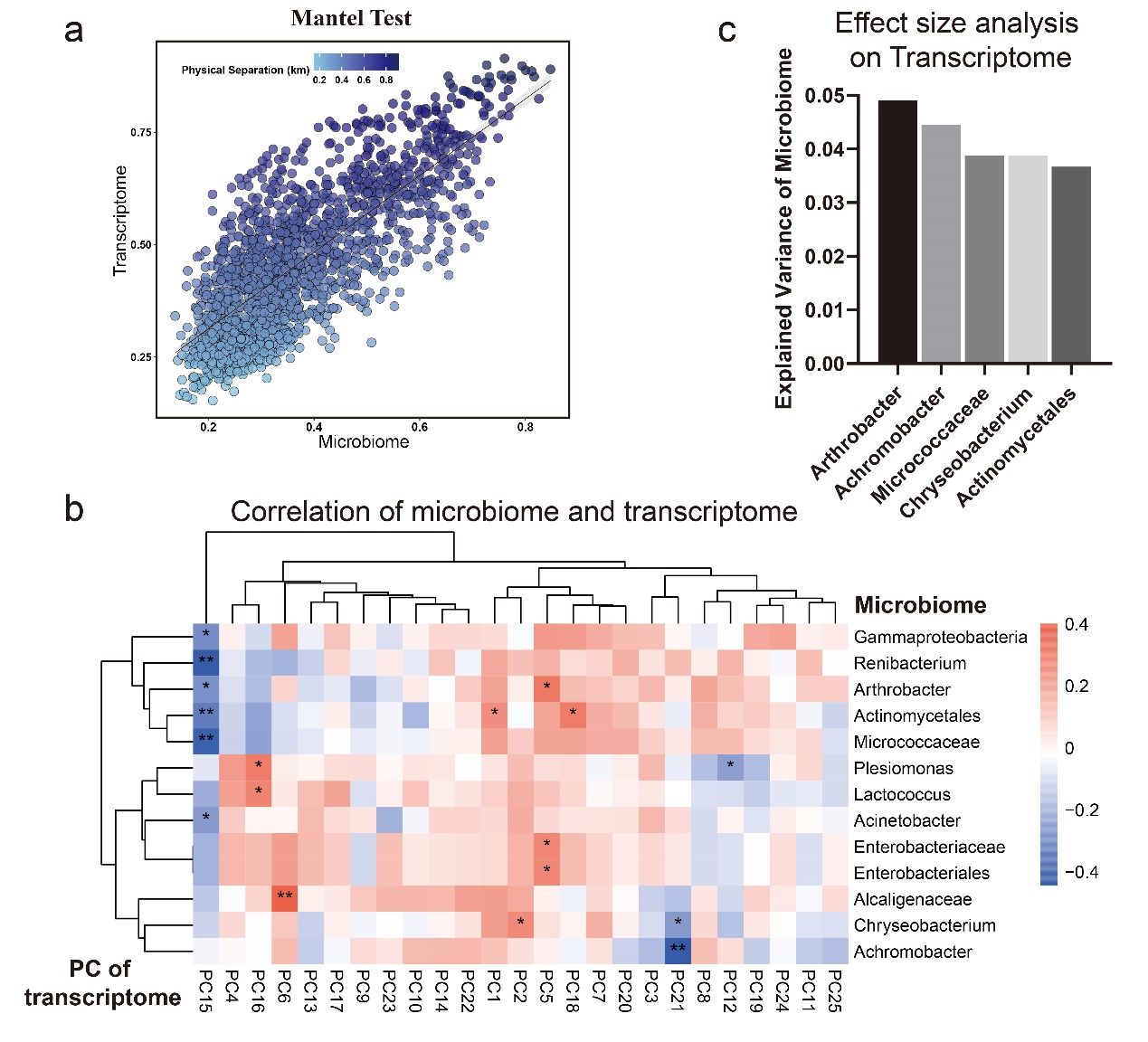


**Fig. S3** **Variance of transcriptome in mAT from CD versus non-CD controls.** **(a)** Mantel test quantifying variance explained between transcriptome and microbiome showed tight coupling of them (Spearman’s correlation, r=0.738, *p*<0.001). **(b)** Heatmap panels showed the spearman correlation coefficient between differential microbes and PCs of transcriptome, for which significant values in correlation test are denoted: *, *p*<0.05; **, *p*<0.01. **c,** Effect size of the differential microbes that contribute significantly to the variance of the transcriptome (*p*<0.05).


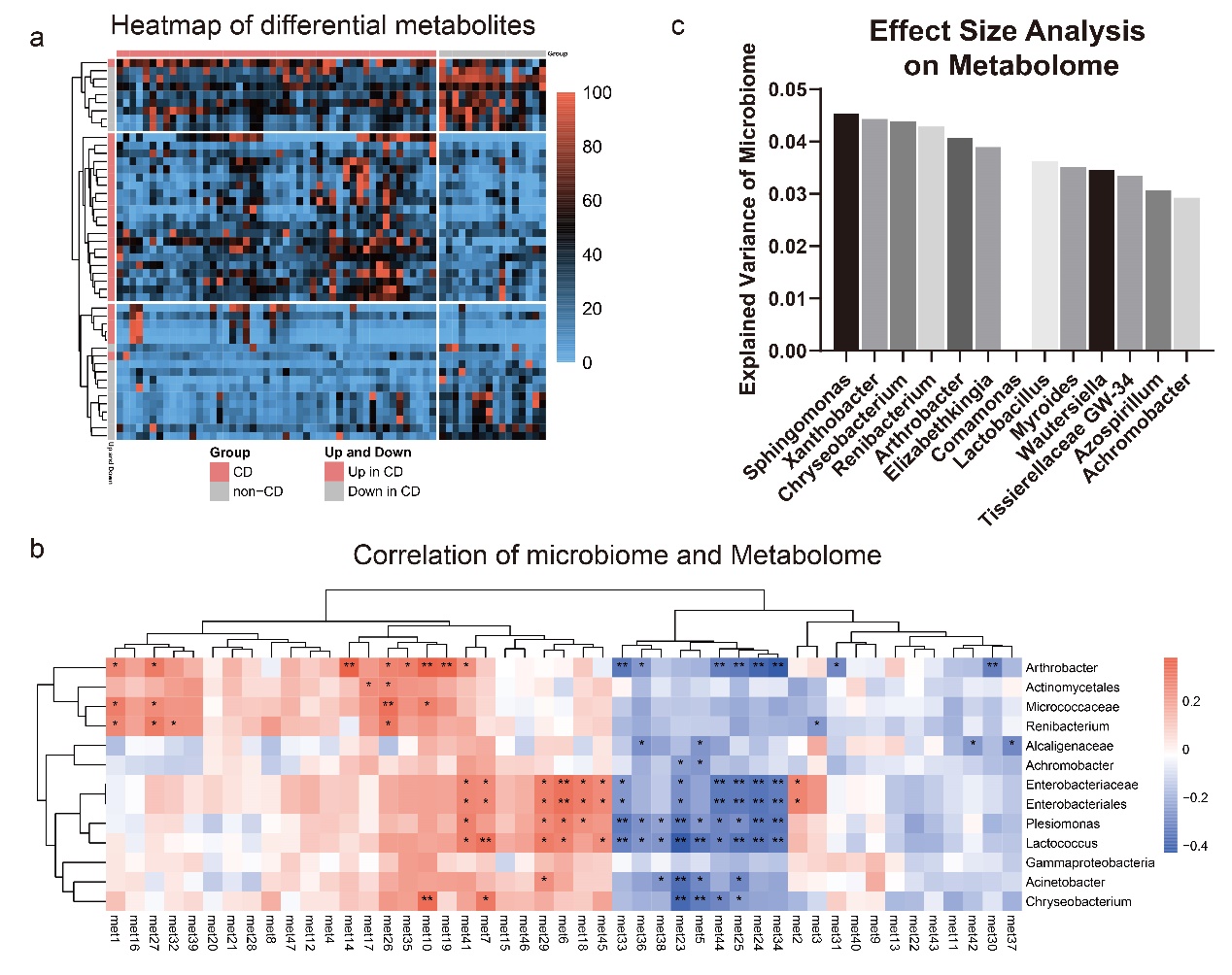


**Fig. S4 Variance of metabolome in mAT from CD versus non-CD controls.** **(a)** Heatmap of the mAT differential metabolites (*p*<0.05, VIP>1) from 48 patients with CD and 16 non-CD controls. Ordering by diagnosis, clustering within diagnosis. **(b)** Heatmap panels show the Spearman correlation coefficient between differential microbes and metabolome, for which significant in correlation test are denoted: *, *p*<0.05; **, *p*<0.01. **(c)** Effect size of the differential microbes that contribute significantly to the variance of the metabolome (*p*<0.05).

**
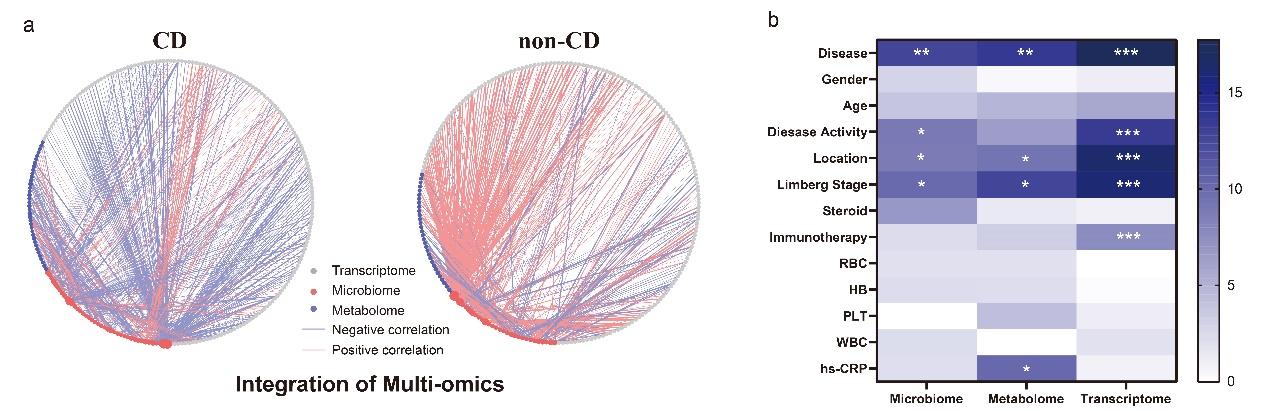
**

**Fig. S5** **Integrated analysis of multi-omics in mAT. (a)** The inter-omics correlation network of all variables for the mesenteric microbiome (red nodes), metabolites (blue nodes) and immune transcripts (gray nodes) of patients with CD and non-CD controls. Nodes indicate omics variables and size of nodes indicate the number of connections. Line between two variables indicates a significant Spearman correlation coefficient at *p*<0.05 (Red, positive correlation; Blue, negative correlation). **(b)** The permutational multivariate analysis of variance (PERMANOVA) shows the effects of clinical variables on the variation of 3 omics datasets of mAT: microbiome, transcriptome and metabolome, for which significant values in test are denoted: *, *p*<0.05; **, *p*<0.01; ***, *p*<0.001.

**
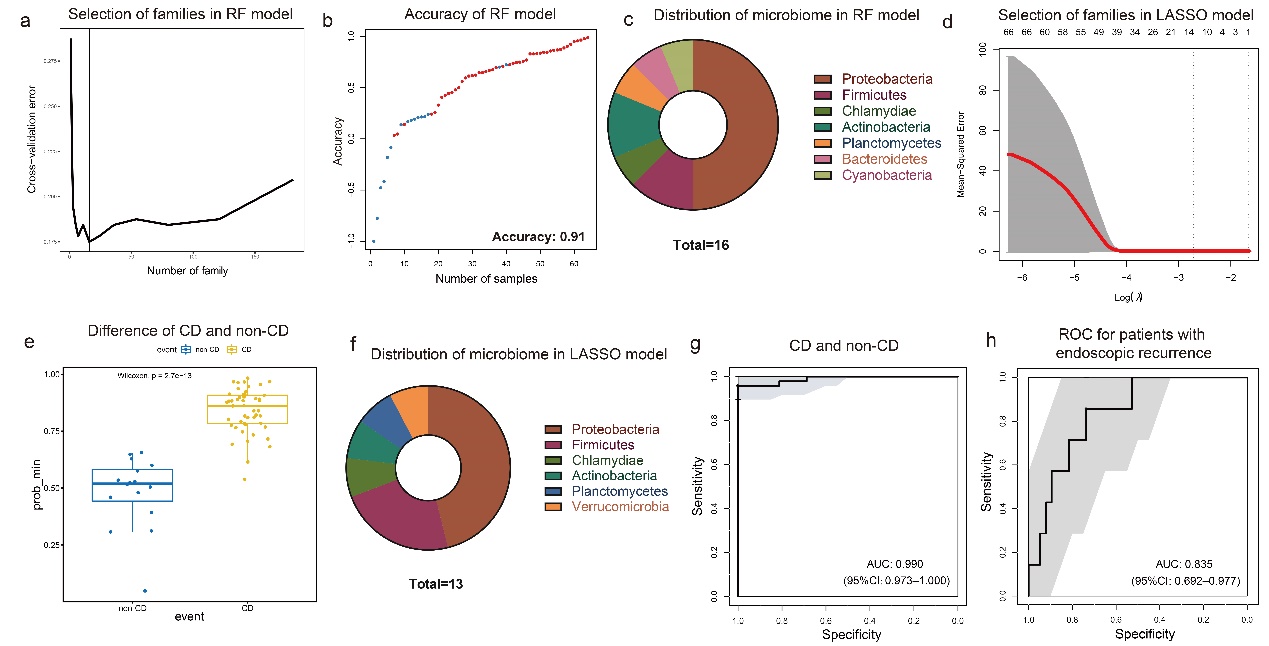
**

**Fig. S6 Potential of mAT microbiome as diagnostic markers for CD and non-CD stratification. (a)** The top 16 mAT bacterial biomarkers were identified by applying Random Forests regression of their relative abundance. **(b)** Accuracy of the random forest (RF) model to classify CD patients and non-CD controls. **(c)** Distribution of mAT microbiome from RF model. **(d)** Optimal value of lambda that minimizes the cross-validation error. **(e)** Using lambda.min as the best lambda, the significantly mAT microbiome difference between CD and non-CD controls based on LASSO logistic regression model. **(f)** Distribution of mAT microbiome from LASSO logistic regression model. **(g)** ROC performance evaluation for LASSO logistic regression model and to distinguish samples from patients with CD and non-CD controls (AUC=0.99). **(h)** ROC analysis of these mAT bacterial biomarkers in LASSO logistic regression model achieved an AUC of 0.835 to classify patients with endoscopic recurrence. The significant values in test are denoted: ns, *p*>0.05; *, *p*<0.05; **, *p*<0.01; ***, *p*<0.001.

**
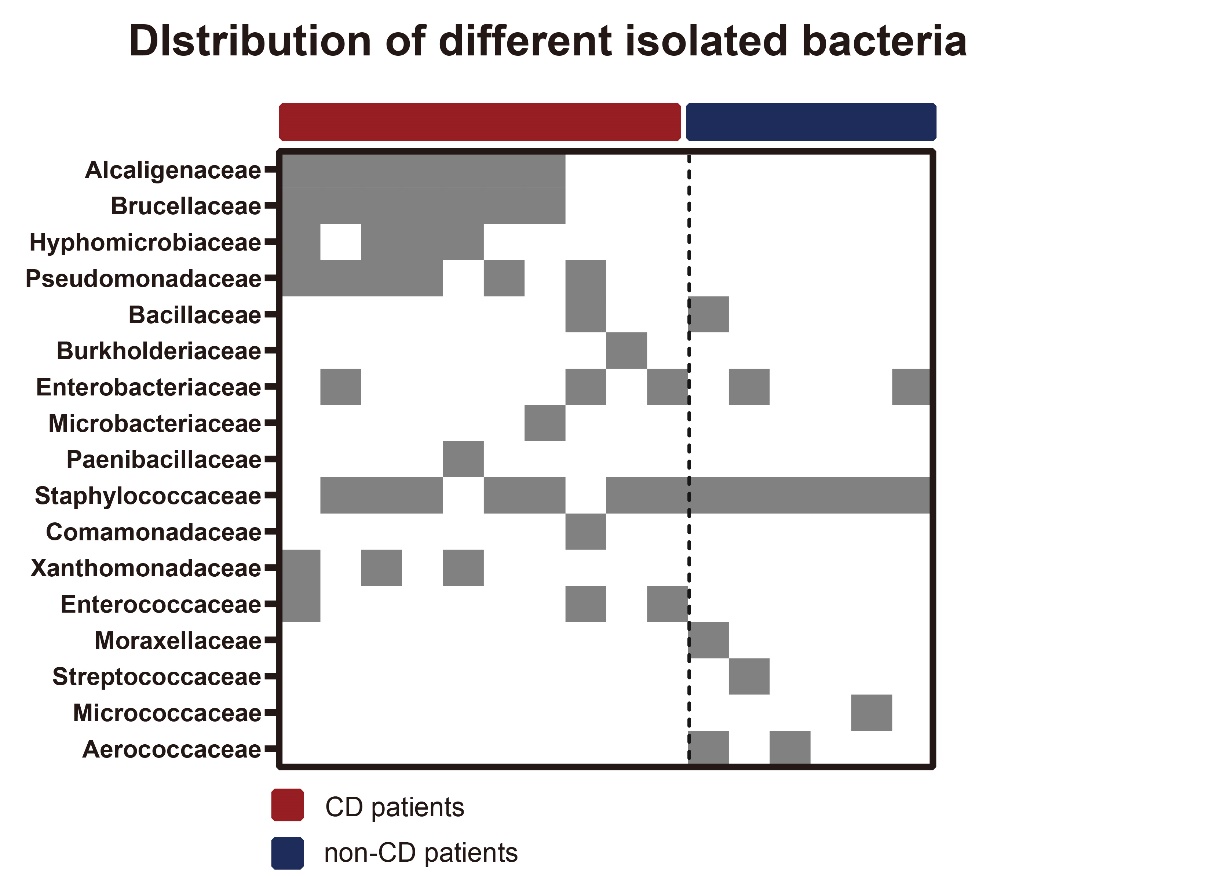
Fig. S7 Distribution of different isolated bacteria in mAT from patients with CD.**


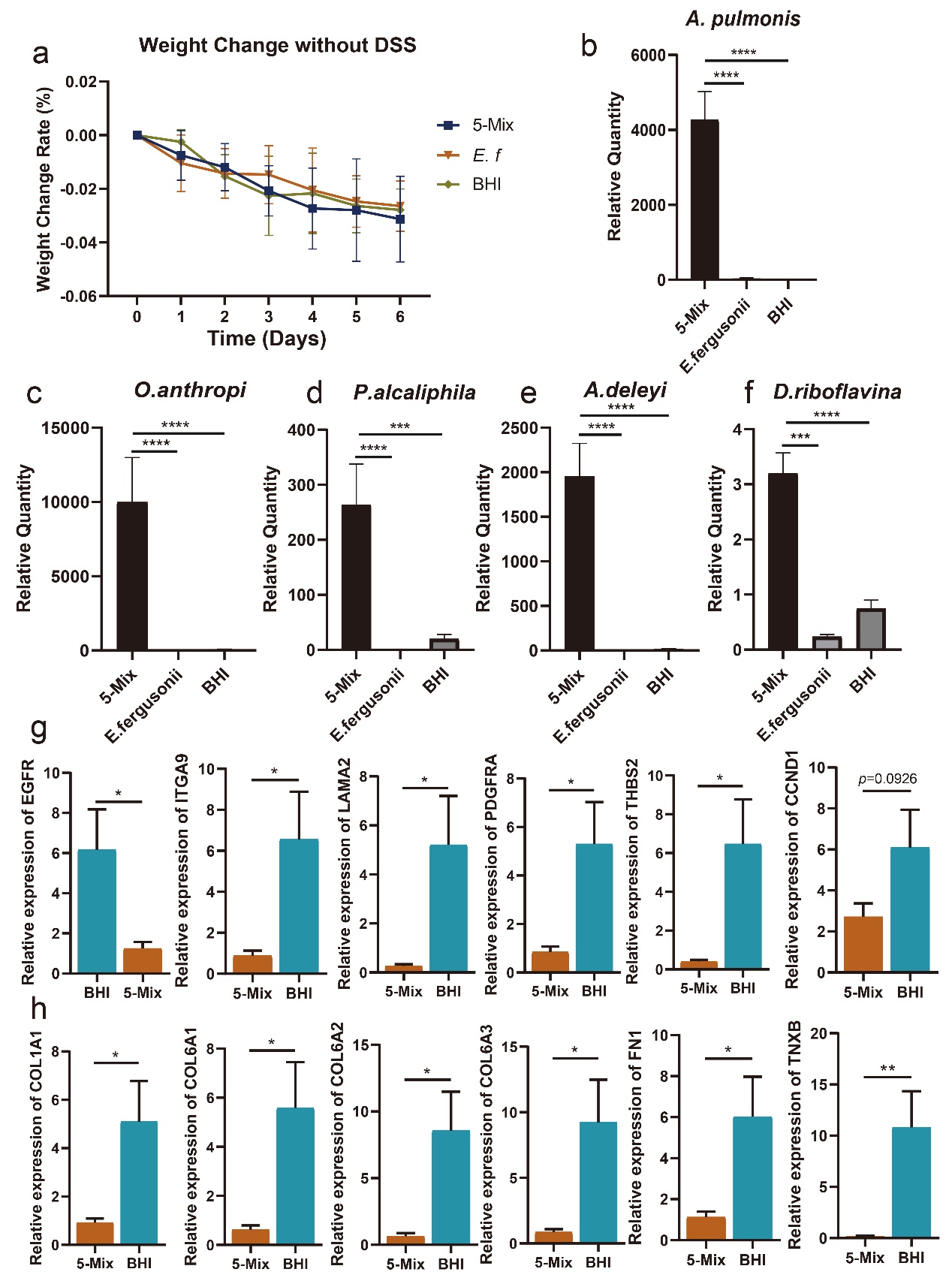


**Fig. S8** **Colonization of different isolated bacteria in mice mediated the re-construction of mAT.** **(a)** The weight loss changes without DSS administration in mice treated with 5-Mix, *E.f* and BHI. **(b-f)** Using quantitative PCR to detect the relative abundance of 5 bacteria in feces from mice treated with 5-Mix, *E.f* and culture medium (BHI). **(g and h)** Several transcripts associated with focal adhesion and ECM-receptor interaction were significantly down-regulated in mice treated with 5-mix bacteria. The significant values in test are denoted: *, *p*<0.05; **, *p*<0.01; ***, *p*<0.001.

**
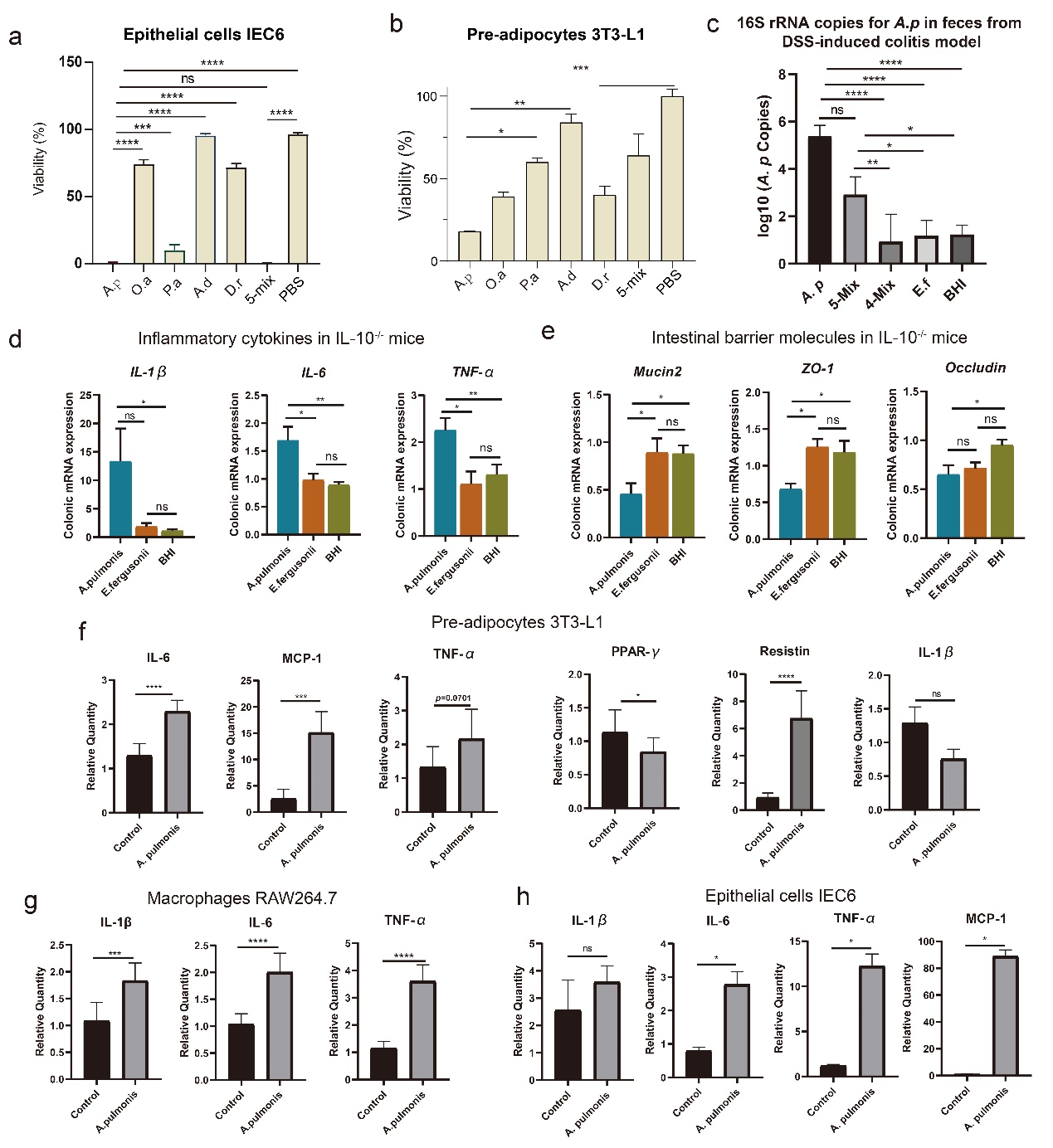
**

**Fig. S9 Pro-inflammatory role of mAT resident *A. pulmonis*.** **(a and b)** Using lactate dehydrogenase (LDH) assay to assess the cell viability after co-culture of epithelial cell line IEC6 (**a)** or pre-adipocyte 3T3-L1 (**b)** with bacteria for 24 hours. **(c)** Quantity of *A. pulmonis* in feces from mice treated with *A. pulmonis*, 5-Mix, 4-Mix, *E.f* and BHI control by quantitative PCR. **(d and e)** Messenger RNA levels of pro-inflammatory cytokines (IL-1β, IL-6 and TNF-α) (**d**) and intestinal barrier molecules (Muc2, ZO-1 and Occludin) (**e**). **(f-h)** Messenger RNA levels of key cytokines and chemokines after co-culture of *A. pulmonis* and different cell lines (**f,** 3T3-L1; **g,** macrophages cell line RAW264.7; **h,** IEC-6). The cells in control group were treated by PBS. The significant values in test are denoted: ns, *p*>0.05; *, *p*<0.05; **, *p*<0.01; ***, *p*<0.001.

**
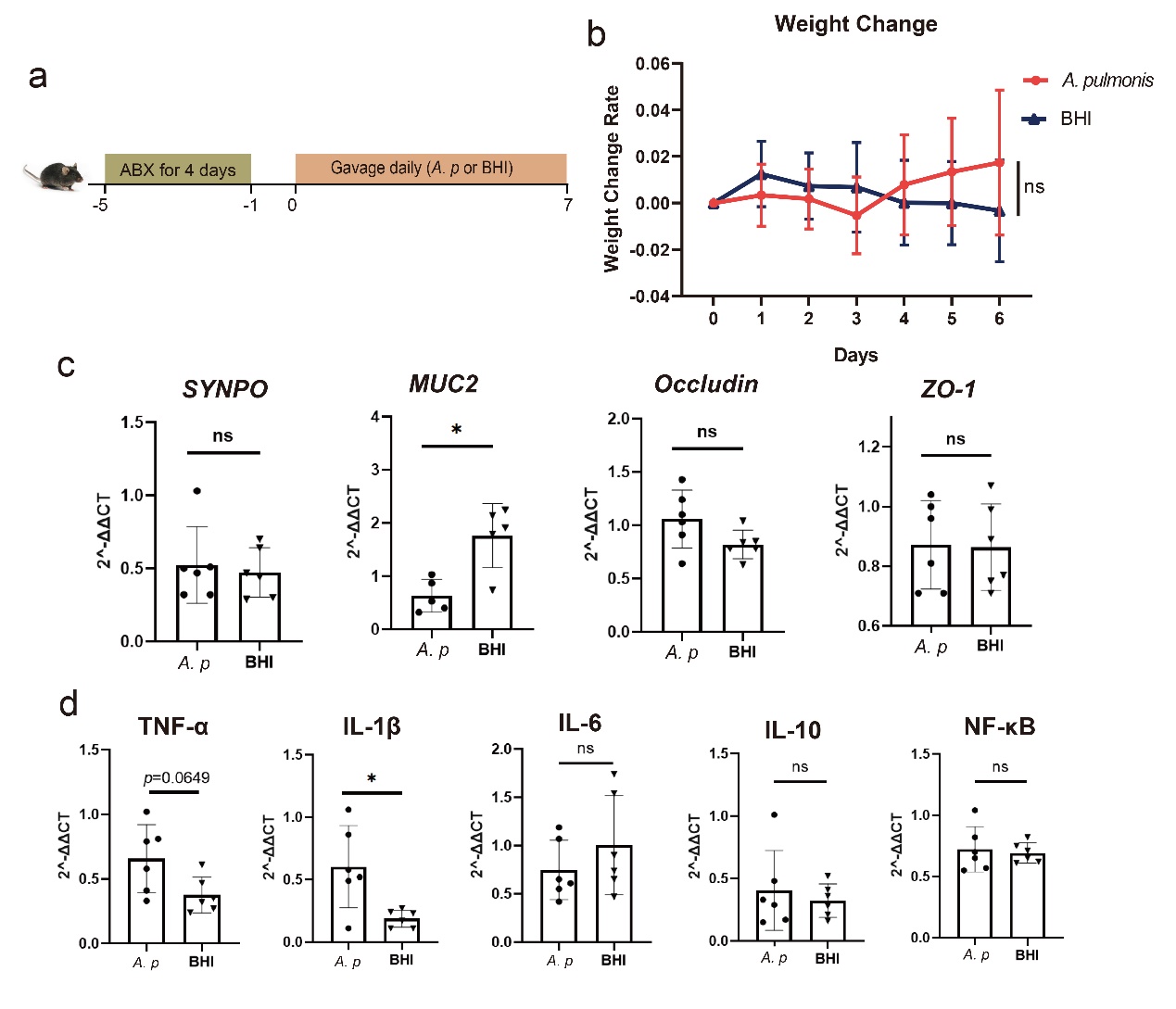
Fig. S10 Intestinal function modulated by *A. pulmonis*. (a)** SPF C57BL/6 mice were treated with antibiotics cocktail for 4 days. One day post antibiotics, the mice were daily orally colonized with *A. pulmonis* (10^9^ CFU/mouse) or BHI for 7 days. **(b)** The weight loss changes without DSS administration between mice treated with *A.pulmonis* and BHI. **(c)** mRNA level of tight junction proteins (*Synpo*, *Muc2*, *Occludin* and *ZO−1*) in the mice colon (n=5 in each group). **(d)** mRNA level of inflammatory molecules (TNFα, IL1β, IL-6, IL-10 and NF-κB) in the colon (n=5 in each group). The significant values in test are denoted: ns, *p*>0.05; *, *p*<0.05.

**
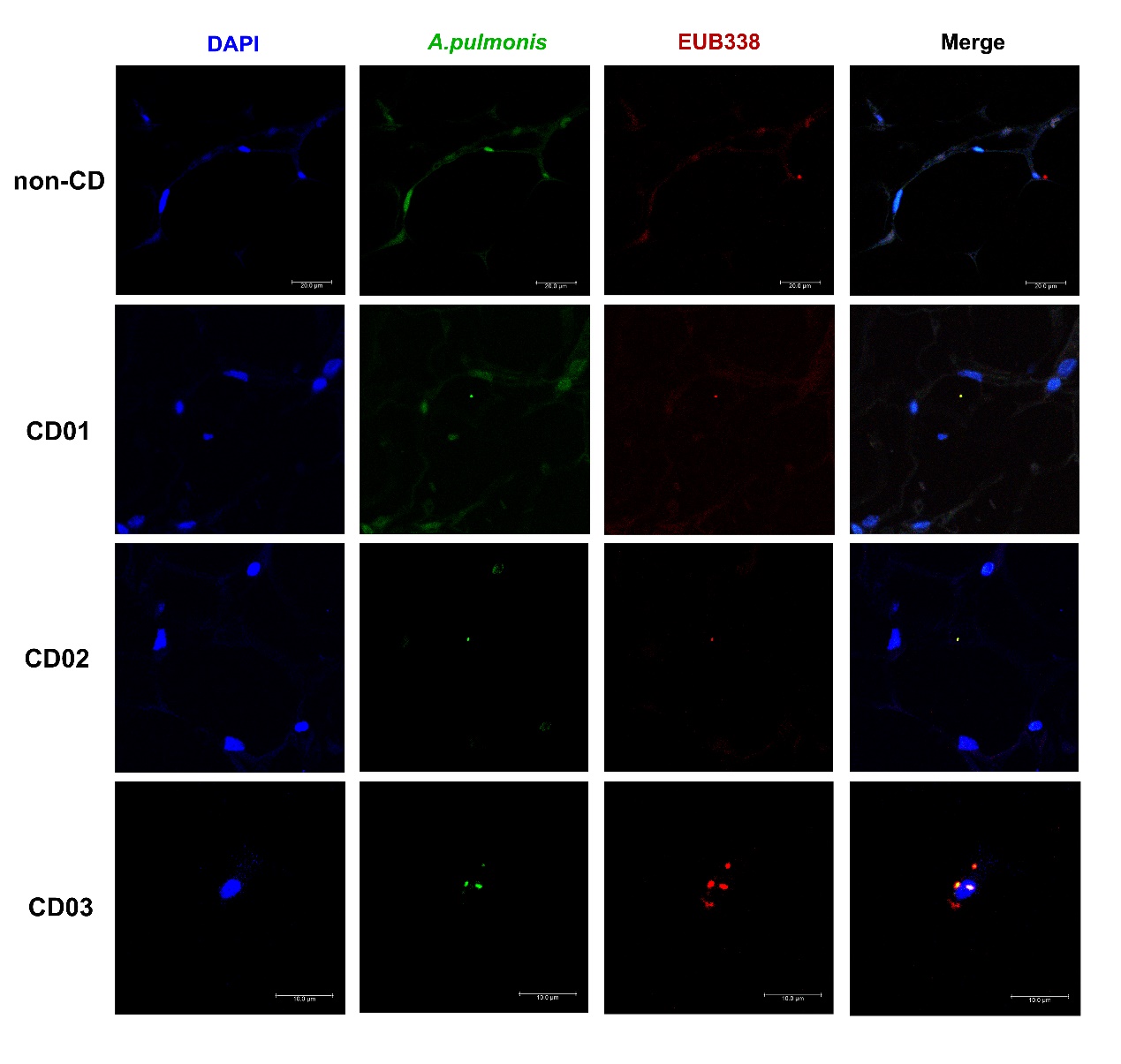
**

**Fig. S11 Visualisation of living bacteria in mAT using FISH.** Representative ﬂuorescence micrographs obtained by FISH using *A.pulmonis* specific probe (in green), EUB338 probe (in red) and DAPI staining (in blue), showing the presence and distribution of *A.pulmonis* in mAT from CD or non-CD controls.

**
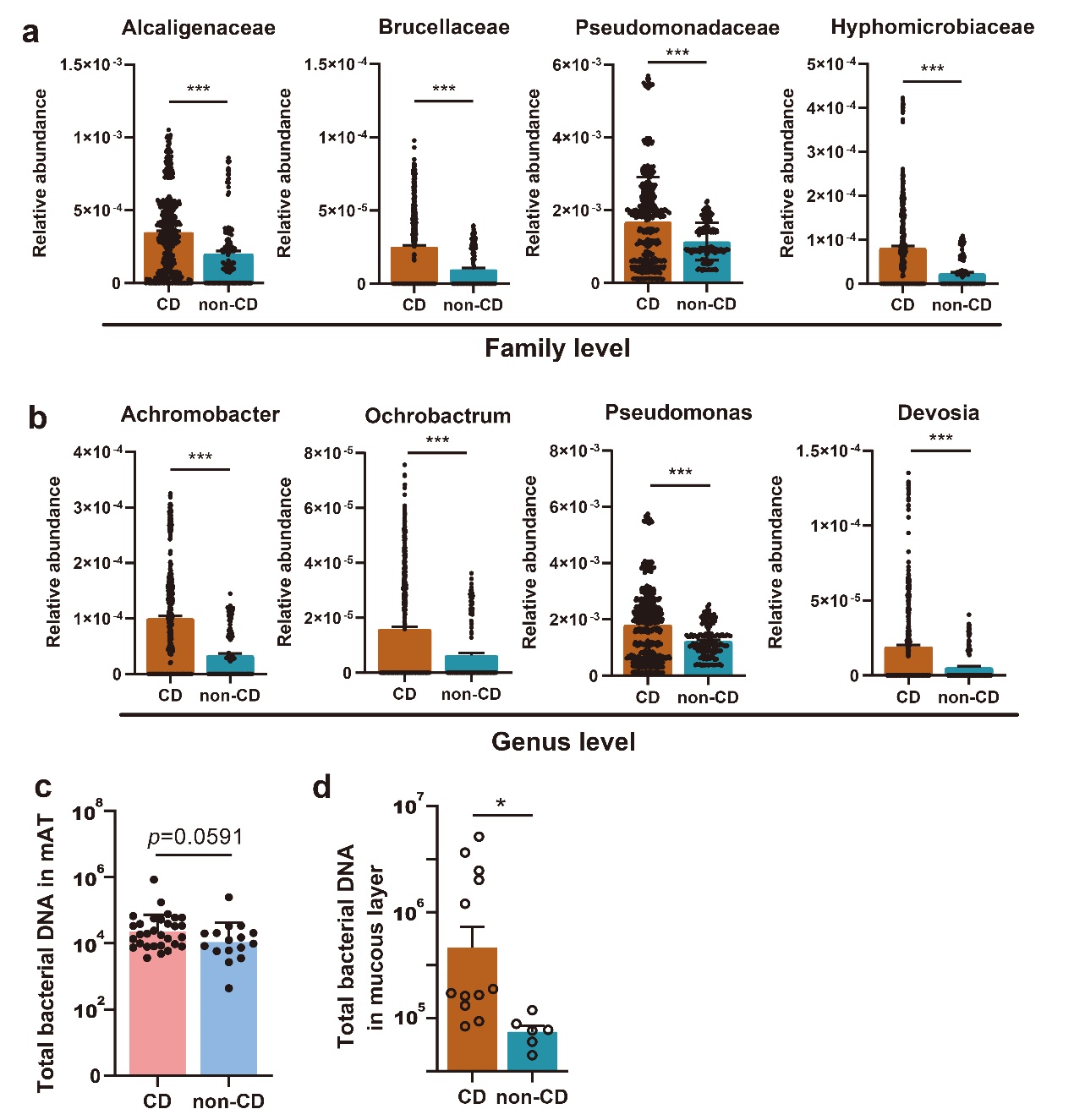
**

**Fig. S12 Bacterial abundance in different clinical cohorts.** **(a)** Relative abundance of fecal Alcaligenaceae, Brucellaceae, Pseudomonadaceae and Hyphomicrobiaceae between CD patients and non-CD controls in RISK cohort. **(b)** Relative abundance of fecal Achromobacter, Ochrobactrum, Pseudomonas and Devosia between CD patients and non-CD controls in RISK cohort. **(c)** Total bacterial DNA in mAT from CD patients and non-CD controls in our clinical cohort. **(d)** Total bacterial DNA in mucous layer from CD patients and non-CD controls in our validation cohort. The significant values in test are denoted: ns, p>0.05; *, p<0.05.

**Table S1. Differential metabolites in mAT between CD and non-CD controls**

| **Differential metabolites** | | | | | |
| --- | --- | --- | --- | --- | --- |
| **id** | **Metabolite** | **Mean in CD** | **Mean in non-CD** | **VIP** | ***p* value** |
| Met01 | (2-propan-2-ylphenyl) n-methylcarbamate | 1.34E-06 | 6.49646E-07 | 1.935930399 | 0.004084137 |
| Met02 | (2r,3r,4s,5s,6r)-2-[(2r,3s,4r,5r,6r)-4,5-dihydroxy-2-(hydroxymethyl)-6-[(2r,3s,4r,5r)-4,5,6-trihydroxy-2-(hydroxymethyl)oxan-3-yl]oxyoxan-3-yl]oxy-6-(hydroxymethyl)oxane-3,4,5-triol | 4.4218E-07 | 2.08623E-07 | 1.404841177 | 0.018215314 |
| Met03 | (2r,3s,4s,5r,6r)-2-(hydroxymethyl)-6-[(2r,3r,4s,5s,6r)-3,4,5-trihydroxy-6-(hydroxymethyl)oxan-2-yl]oxyoxane-3,4,5-triol | 0.00010902 | 6.96384E-05 | 1.506538985 | 0.025151228 |
| Met04 | (2s)-2-amino-4-(2-aminophenyl)-4-oxobutanoic acid | 1.3605E-06 | 4.12046E-07 | 2.392018772 | 0.000594857 |
| Met05 | (z)-octadec-9-enoic acid | 2.6593E-05 | 4.14881E-05 | 2.299077789 | 0.000183225 |
| Met06 | 1-[(3,4-dimethoxyphenyl)methyl]-6,7-dimethoxy-2-methyl-3,4-dihydro-1h-isoquinoline | 0.00013278 | 4.84942E-05 | 1.328413977 | 0.022676806 |
| Met07 | 1h-pyridin-2-one | 1.552E-06 | 9.89317E-07 | 1.745716419 | 0.04891988 |
| Met08 | 2-(1h-imidazol-5-yl)ethanamine | 0.00045369 | 0.000295382 | 1.805410315 | 0.039155924 |
| Met09 | 2-(3-hydroxyphenyl)acetic acid | 1.6637E-05 | 2.67384E-05 | 2.137639229 | 0.013050609 |
| Met10 | 2,6-dimethylpyridine | 1.3777E-05 | 1.23655E-05 | 1.049991316 | 0.022332611 |
| Met11 | 2-furancarboxaldehyde | 2.1071E-05 | 2.85278E-05 | 2.027220292 | 0.006623151 |
| Met12 | 2-methoxycanthin-6-one | 3.4565E-06 | 2.42365E-06 | 1.866526838 | 0.032664706 |
| Met13 | 3-hydroxy-5-(hydroxymethyl)-2-methylpyridine-4-carbaldehyde | 1.3437E-05 | 1.64655E-05 | 1.715354682 | 0.031987988 |
| Met14 | 4,5-dimethyloxazole | 5.4274E-05 | 4.08439E-05 | 1.312980876 | 0.00901467 |
| Met15 | 5-ethyl-2-hexyl-4-methyloxazole | 1.1858E-06 | 7.28098E-07 | 2.577086508 | 0.000958755 |
| Met16 | 9-[(2r,3r,4s,5r)-3,4-dihydroxy-5-(hydroxymethyl)oxolan-2-yl]-3h-purine-2,6-dione | 2.7379E-06 | 1.16606E-06 | 2.110093918 | 0.000633743 |
| Met17 | acetamidopropanal | 0.00074842 | 5.86053E-06 | 2.225040562 | 5.92208E-09 |
| Met18 | all-trans-carophyll yellow | 1.8455E-06 | 1.8417E-07 | 1.532482287 | 0.023060927 |
| Met19 | alpha-butyl-omega-hydroxypoly(oxyethylene) poly(oxypropylene) | 0.00166448 | 0.001421787 | 1.433289495 | 0.015143848 |
| Met20 | choline | 0.00347708 | 0.002794387 | 2.114281912 | 0.017724436 |
| Met21 | d-4'-phosphopantothenate | 1.4566E-06 | 3.99353E-07 | 2.438299385 | 3.96343E-06 |
| Met22 | deterrol stearate | 5.7903E-06 | 8.01913E-06 | 2.210400744 | 0.014699856 |
| Met23 | d-maltose | 3.9362E-06 | 5.62581E-06 | 2.025569221 | 0.013464244 |
| Met24 | elaidic carnitine | 5.4055E-05 | 0.000120005 | 2.139387499 | 0.00028723 |
| Met25 | epoxy-hexobarbital | 5.9273E-06 | 1.21848E-05 | 3.011103591 | 0.014809958 |
| Met26 | fad | 1.1635E-06 | 7.93893E-07 | 2.362005427 | 0.005028376 |
| Met27 | fentanyl | 7.2212E-06 | 5.23626E-06 | 1.611903912 | 0.036831844 |
| Met28 | fludarabine | 3.8105E-06 | 2.5865E-06 | 1.321238043 | 0.024087211 |
| Met29 | fomepizole | 3.9424E-07 | 9.43376E-08 | 1.041460357 | 0.020190514 |
| Met30 | glutamylphenylalanine | 3.1399E-07 | 8.4595E-07 | 3.024816767 | 5.56451E-12 |
| Met31 | hexan-1-amine | 4.1158E-06 | 9.37223E-06 | 2.425186307 | 2.51657E-12 |
| Met32 | hordatine a glucoside | 1.2674E-06 | 2.78618E-07 | 2.404327732 | 0.000464036 |
| Met33 | linoleyl carnitine | 2.4252E-05 | 5.16296E-05 | 2.039294866 | 0.008658612 |
| Met34 | l-palmitoylcarnitine | 5.7909E-05 | 0.000109874 | 1.900585609 | 0.014637414 |
| Met35 | lysopc(18:2(9z,12z)) | 0.00031948 | 0.000226408 | 1.68222916 | 0.000539119 |
| Met36 | lysopc(22:6(4z,7z,10z,13z,16z,19z)) | 2.1628E-06 | 8.76409E-06 | 2.147478528 | 0.008863019 |
| Met37 | lysope(22:6(4z,7z,10z,13z,16z,19z)/0:0) | 1.9174E-05 | 3.35101E-05 | 1.426444341 | 0.022073371 |
| Met38 | mannitol | 8.2523E-07 | 1.05076E-06 | 1.42673457 | 0.007070115 |
| Met39 | niacinamide | 0.00075355 | 0.00057976 | 2.062557278 | 0.017559316 |
| Met40 | oseltamivir | 2.4406E-05 | 2.93884E-05 | 1.378686691 | 0.020040354 |
| Met41 | petroselinic acid | 1.0827E-06 | 2.17946E-07 | 3.203984626 | 8.07432E-09 |
| Met42 | phytanic acid | 8.1834E-06 | 9.29019E-06 | 1.134157453 | 0.035608321 |
| Met43 | polypropylene glycol (m w 1,200-3,000) | 5.0283E-06 | 5.96989E-06 | 1.970902473 | 0.000162884 |
| Met44 | stearoylcarnitine | 2.4526E-05 | 4.80916E-05 | 1.965147399 | 0.001151103 |
| Met45 | tazobactam | 1.2979E-06 | 7.43239E-07 | 1.304002016 | 0.01384823 |
| Met46 | tetradecanoic acid | 1.6542E-06 | 1.0405E-06 | 1.874957235 | 0.031106598 |
| Met47 | valyl-proline | 7.9896E-07 | 3.67914E-07 | 1.613079586 | 0.005238489 |

**Table S2. Correlation analysis of microbiome, transcriptome and metabolome in CD and non-CD cohorts.**

|  | **CD** | | **non-CD** | | ***p* value** |
| --- | --- | --- | --- | --- | --- |
|  | **Num.** | **Percentage (%)** | **Num.** | **Percentage (%)** |  |
| **Positive correlation** | 168 | 27.91 | 302 | 72.08 | **<0.001** |
| **Negative correlation** | 434 | 72.09 | 117 | 27.92 |  |
| **Total** | 602 | 100 | 419 | 100 |  |

**Table S3. Summary on statistics of the host properties and clinical indexes from CD and non-CD cohorts.**

|  | | **Patients with CD**  **(n=48)** | | **non-CD patient**  **(n=16)** | | ***p* value** | |
| --- | --- | --- | --- | --- | --- | --- | --- |
|  |  |  |  |  |  |  |  |
|  |  |  |  |  |  |  |  |
| Age (y) | | 32.92±9.27 | | 69.5±16.82 | | <0.001 | |
| Sex (female, %) | | 37.5 | | 56.3 | | 0.2461 | |
| Disease duration (y) | | 5.42±4.63 | |  | |  | |
| Activity (%) | |  | |  | |  | |
| Active | | 50 | |  | |  | |
| Remission | | 50 | |  | |  | |
| Location (%) | |  | |  | |  | |
| L1/L2/L3 | | 14.58/4.17/81.25 | |  | |  | |
| Limberg stage (%) | |  | |  | |  | |
| I/II/III/IV | | 2.08/52.08/33.33/12.50 | |  | |  | |
| WBC (10^9^/L) | | 6.02±2.98 | | 6.29±1.58 | | 0.7315 | |
| RBC (10^12^/L) | | 4.60±0.70 | | 4.03±0.70 | | 0.0071 | |
| PLT (10^9^/L) | | 268.87±88.86 | | 271.38±71.99 | | 0.9192 | |
| Hb (g/L) | | 123.65±18.85 | | 116.06±24.87 | | 0.2042 | |
| K (mmol/L) | | 4.02±0.84 | | 4.01±0.30 | | 0.9480 | |
| Na (mmol/L) | | 139.99±2.01 | | 140.05±1.87 | | 0.9255 | |
| Cl (mmol/L) | | 104.05±2.41 | | 105.37±3.47 | | 0.0966 | |
| Ca (mmol/L) | | 2.30±0.17 | | 2.29±0.12 | | 0.7651 | |
| Cr (μmol/L) | | 65.54±16.22 | | 76.77±19.68 | | 0.0282 | |
| ALT (U/L) | | 29.13±24.91 | | 22.17±20.24 | | 0.3163 | |
| AST (U/L) | | 26.62±13.31 | | 24.03±15.30 | | 0.5189 | |
| ALB (g/L) | | 39.81±5.91 | | 39.63±3.97 | | 0.9124 | |
| Treatment (%) | |  | |  | |  | |
| Mesalazine | | 43.75 | |  | |  | |
| Steroids | | 35.42 | |  | |  | |
| Immunomodulators | | 66.67 | |  | |  | |
| EEN | | 93.75 | |  | |  | |
| Background disease | | Crohn’s disease | | Colorectal cancer | |  | |
| Rutgeerts score, n (%) | |  | |  | |  | |
| i0-i1 | | 26 (54.17) | |  | |  | |
| i2-i4 | | 19 (39.58) | |  | |  | |
| no record | | 3 (6.25) | |  | |  | |

WBC, white blood cell; RBC, red blood cell; PLT, platelet; Hb, hemoglobin; Cr, creatinine; ALT, alanine aminotransferase; AST, aspartate aminotransferase; ALB, albumin; EEN, exlusive enteral nutrition

**Table S4. Summary on statistics of the clinical indexes from patients with and without endoscopic recurrence.**

| **Rutgeerts score** | **Non-recurrence**  **(i0-i1)** | **Endoscopic recurrence**  **(i2-i4)** | ***P* value** |
| --- | --- | --- | --- |
| Patients (n) | 26 | 19 | / |
| ESR (mm/hr) | 21.55±18.69 | 22.28±18.57 | 0.818 |
| WBC (109/L) | 5.21±1.56 | 5.17±1.58 | 0.059 |
| CRP (mg/L) | 4.19±6.40 | 4.28±6.45 | 0.063 |
| Duration (month) | 10.78±6.33 | 10.38±6.17 | 0.807 |

ESR, erythrocyte sedimentation rate; WBC, white blood cell; CRP, C-reactive protein

**Table S5. Summary information of 174 isolated colonies in this study.**

| **Culture and Identification** | | | | |
| --- | --- | --- | --- | --- |
| **Phylum** | **Family** | **Strains** | **Colonies** | **Notes** |
| ***Proteobacteria*** | ***Alcaligenaceae*** | ***Achromobacter pulmonis*** | **32** | ***A.pulmonis*** |
| ***Proteobacteria*** | ***Brucellaceae*** | ***Ochrobactrum anthropi*** | **21** | ***O.anthropi*** |
| *Firmicutes* | *Enterococcaceae* | *Enterococcus durans* | 16 |  |
| *Proteobacteria* | *Enterobacteriaceae* | *Shigella sonnei* | 16 |  |
| ***Proteobacteria*** | ***Pseudomonadaceae*** | ***Pseudomonas alcaliphila*** | **14** | ***P.alcaliphila*** |
| ***Proteobacteria*** | ***Alcaligenaceae*** | ***Achromobacter deleyi*** | **11** | ***A.deleyi*** |
| *Firmicutes* | *Staphylococcaceae* | *Staphylococcus hominis* | 8 |  |
| ***Proteobacteria*** | ***Hyphomicrobiaceae*** | ***Devosia riboflavina*** | **6** | ***D.riboflavina*** |
| *Proteobacteria* | *Enterobacteriaceae* | *Escherichia fergusonii* | 6 |  |
| *Firmicutes* | *Enterococcaceae* | *Enterococcus faecalis* | 5 |  |
| *Proteobacteria* | *Comamonadaceae* | *Pseudacidovorax intermedius* | 5 |  |
| *Proteobacteria* | *Enterobacteriaceae* | *Klebsiella variicola* | 4 |  |
| *Proteobacteria* | *Enterobacteriaceae* | *Shigella flexneri* | 4 |  |
| *Proteobacteria* | *Xanthomonadaceae* | *Stenotrophomonas maltophilia* | 4 |  |
| *Proteobacteria* | *Enterobacteriaceae* | *Klebsiella pneumoniae* | 2 |  |
| *Firmicutes* | *Staphylococcaceae* | *Staphylococcus cohnii* | 2 |  |
| *Firmicutes* | *Staphylococcaceae* | *Staphylococcus haemolyticus* | 2 |  |
| *Firmicutes* | *Staphylococcaceae* | *Staphylococcus hominis* | 2 |  |
| *Proteobacteria* | *Alcaligenaceae* | *Achromobacter denitrificans* | 1 |  |
| *Firmicutes* | *Bacillaceae* | *Bacillus niabensis* | 1 |  |
| *Firmicutes* | *Bacillaceae* | *Bacillus oceanisediminis* | 1 |  |
| *Firmicutes* | *Bacillaceae* | *Bacillus pseudomycoides* | 1 |  |
| *Firmicutes* | *Bacillaceae* | *Bacillus subtilis* | 1 |  |
| *Firmicutes* | *Bacillaceae* | *Bacillus wiedmannii* | 1 |  |
| *Actinobacteria* | *Microbacteriaceae* | *Microbacterium testaceum* | 1 |  |
| *Firmicutes* | *Paenibacillaceae* | *Paenibacillus barcinonensis* | 1 |  |
| *Proteobacteria* | *Burkholderiaceae* | *Paraburkholderia fungorum* | 1 |  |
| *Proteobacteria* | *Pseudomonadaceae* | *Pseudomonas chengduensis* | 1 |  |
| *Proteobacteria* | *Pseudomonadaceae* | *Pseudomonas entomophila* | 1 |  |
| *Firmicutes* | *Staphylococcaceae* | *Staphylococcus epidermidis* | 1 |  |
| *Firmicutes* | *Staphylococcaceae* | *Staphylococcus hominis* | 1 |  |
| *Proteobacteria* | *Xanthomonadaceae* | *Stenotrophomonas maltophilia* | 1 |  |
| **Total** |  |  | **174** |  |

**Table S6. Primer sequences used in this study.**

| **Origin** | **Primers** | **Forward** | **Reverse** |
| --- | --- | --- | --- |
| **Mouse** | **IL-1β** | GCTGAAAGCTCTCCACCTCA | GCTTGGGATCCACACTCTCC |
|  | **IL-6** | CTCTGCAAGAGACTTCCATCCA | GACAGGTCTGTTGGGAGTGG |
|  | **TNF-⍺** | GCCTCTTCTCATTCCTGCTTG | CTGATGAGGGAGGCCATT |
|  | **NF-κ B** | GAAATTCCCTGATCCAGACAAAAAC | ATCACTTCAATGGCCTCTGTGTAG |
|  | **IL-10** | ATAAACTGCACCCCACTTCCCA | TGGACCATCTTCACTACGGG |
|  | **MCP-1** | CATAGCAGCCACCTTCATTCC | TCTGCACTGAGATCTTCCTATTGG |
|  | **Resistin** | GTACCCACGGGATGAAGAACC | GCAGAGCCACAGGAGCAG |
|  | **PPAR-γ** | TCAGCTCTGTGGACCTCTCC | ACCCTTGCATCCTTCACAAG |
|  | **ZO-1** | TCATCCCAAATAAGAACAGAGC | GAAGAACAACCCTTTCATAAGC |
|  | **Occludin** | CTTTGGCTACGGAGGTGGCTAT | CTTTGGCTGCTCTTGGGTCTG |
|  | **Synpo** | ATGGAGGGGTACTCAGAGGAG | CTCTCGGTTTTGGGACAGGTG |
|  | **Muc2** | GCCAGATCCCGAAACCA | TATAGGAGTCTCGGCAGTCA |
|  | **Gapdh** | TGAAGCAGGCATCTGAGGG | CGAAGGTGGAAGAGTGGGAG |
| **Rat** | **MCP-1** | TGAACCTGGAGGCTACAGTGAA | GGCCTCGGCGTTTGG |
|  | **IL-1β** | CACCTCTCAAGCAGAGCACAG | GGGTTCCATGGTGAAGTCAAC |
|  | **IL-6** | AAAGAGTTGTGCAATGGCAATTCT | CAGTGCATCATCGCTGTTCATACA |
|  | **TNF-⍺** | ACTGAACTTCGGGGTGATTG | GCTTGGTGGTTTGCTACGAC |
|  | **Gapdh** | GGCATTGCTCTCAATGACAA | AGGGCCTCTCTCTTGCTCTC |
| ***A.pulmonis*** | | GTCGAACGGTAACACGGACT | GTGTCTCAGTCCCAGTGTGG |
| ***O.anthropi*** | | ACGGTCGCATTGGTTACACT | TGTCGCCCGGATCGAAATAG |
| ***P.alcaliphila*** | | CGCACTGCAACAAGCATTGA | CCAGACCGGAAAAGCCGTAA |
| ***A.deleyi*** | | TGACGTCAAGTCCTCATGGC | ATCCTACCGTGGTAATCGCC |
| ***D.riboflavina*** | | ATGATCGTGCTTTCCATCGC | GCACGACATGAATCTGGCTT |
